# Expansion of wastewater-based disease surveillance to improve health equity in California’s Central Valley: sequential shifts in case-to-wastewater and hospitalization-to-wastewater ratios

**DOI:** 10.3389/fpubh.2023.1141097

**Published:** 2023-06-30

**Authors:** Krystin F. Kadonsky, Colleen C. Naughton, Mirjana Susa, Rachel Olson, Guadalupe L. Singh, Maria L. Daza-Torres, J. Cricelio Montesinos-López, Yury Elena Garcia, Maftuna Gafurova, Adam Gushgari, John Cosgrove, Bradley J. White, Alexandria B. Boehm, Marlene K. Wolfe, Miriam Nuño, Heather N. Bischel

**Affiliations:** ^1^Department of Civil and Environmental Engineering, University of California, Merced, Merced, CA, United States; ^2^Department of Public Health Sciences, University of California, Davis, Davis, CA, United States; ^3^Department of Civil and Environmental Engineering, University of California, Davis, Davis, CA, United States; ^4^Eurofins Environment Testing US, West Sacramento, CA, United States; ^5^Verily Life Sciences, South San Francisco, CA, United States; ^6^Department of Civil & Environmental Engineering, School of Engineering and Doerr School of Sustainability, Stanford University, Stanford, CA, United States; ^7^Gangarosa Department of Environmental Health, Rollins School of Public Health, Emory University, Atlanta, GA, United States

**Keywords:** SARS-CoV-2, COVID-19, environmental surveillance, wastewater clinical case ratios, health metrics

## Abstract

**Introduction:**

Over a third of the communities (39%) in the Central Valley of California, a richly diverse and important agricultural region, are classified as disadvantaged—with inadequate access to healthcare, lower socio-economic status, and higher exposure to air and water pollution. The majority of racial and ethnic minorities are also at higher risk of COVID-19 infection, hospitalization, and death according to the Centers for Disease Control and Prevention. Healthy Central Valley Together established a wastewater-based disease surveillance (WDS) program that aims to achieve greater health equity in the region through partnership with Central Valley communities and the Sewer Coronavirus Alert Network. WDS offers a cost-effective strategy to monitor trends in SARS-CoV-2 community infection rates.

**Methods:**

In this study, we evaluated correlations between public health and wastewater data (represented as SARS-CoV-2 target gene copies normalized by pepper mild mottle virus target gene copies) collected for three Central Valley communities over two periods of COVID-19 infection waves between October 2021 and September 2022. Public health data included clinical case counts at county and sewershed scales as well as COVID-19 hospitalization and intensive care unit admissions. Lag-adjusted hospitalization:wastewater ratios were also evaluated as a retrospective metric of disease severity and corollary to hospitalization:case ratios.

**Results:**

Consistent with other studies, strong correlations were found between wastewater and public health data. However, a significant reduction in case:wastewater ratios was observed for all three communities from the first to the second wave of infections, decreasing from an average of 4.7 ± 1.4 over the first infection wave to 0.8 ± 0.4 over the second.

**Discussion:**

The decline in case:wastewater ratios was likely due to reduced clinical testing availability and test seeking behavior, highlighting how WDS can fill data gaps associated with under-reporting of cases. Overall, the hospitalization:wastewater ratios remained more stable through the two waves of infections, averaging 0.5 ± 0.3 and 0.3 ± 0.4 over the first and second waves, respectively.

## 1. Introduction

California experienced approximately ten million infections and 100,000 deaths between January 2020 and December 2022 from the coronavirus disease (COVID-19) pandemic, caused by severe acute respiratory syndrome coronavirus 2 (SARS-CoV-2) (1). Individuals infected with SARS-CoV-2 often shed viral particles and associated ribonucleic acid (RNA) in their feces regardless of experiencing gastrointestinal symptoms (2). Fecal shedding dominates the total SARS-CoV-2 RNA load in community-level wastewater surveillance compared to other viral shedding routes such as respiratory fluids, saliva, and urine (3). The strong presence of SARS-CoV-2 RNA in wastewater settled solids allows for wastewater-based disease surveillance (WDS) to be utilized as a highly sensitive method to track the environmental persistence and community transmissivity of the virus (along with other highly infectious diseases) (4–6).

WDS involves collection of community-pooled samples of uninfected, asymptomatic, pre-symptomatic, and symptomatic infected individuals from centralized wastewater treatment plants (WWTPs), or, less often, from sewer collection systems (7). Traditional epidemiological monitoring through clinical surveillance is dependent upon infected individuals seeking clinical testing and the availability of clinical tests. WDS offers a less biased mechanism to track viral outbreaks and community infections and can serve as an early indicator of increased COVID-19 community transmission by detecting the virus before symptom onset (8).

Communities around the world rapidly implemented WDS early on in the pandemic. COVIDPoops19, a global ArcGIS dashboard, monitored the growth of WDS implementation since September 2020, including in California (9). Approximately 90% of California’s population is serviced by publicly owned centralized WWTPs, with the remaining population serviced by onsite septic systems (10). As of August 2021, 48 of 384 WWTPs in California were monitoring for SARS-CoV-2 RNA in their communities. The majority of WDS programs (70%), at the time, were located in urban areas of Coastal and Southern California. Only 30% of WDS programs were located in rural areas of Central and Northern California (11). Most WWTPs in California are small or moderate in size (10), and more likely to lack the necessary resources and funding to support WDS programs.

Healthy Central Valley Together (HCVT) was launched in the summer of 2021 to expand WDS as a public health tool for greater health equity in rural and disadvantaged communities (DACs). The Central Valley is located in the heart of California, encompassing communities in nineteen counties (12). Over a third of the Central Valley communities (39%) are classified as DACs by CalEnviroScreen 4.0, compared to 31% of communities in California that are DACs overall (13). Approximately 40% of the population in the Central Valley are located within a DAC, compared to 29% of the overall population in California that live in a DAC (13). Of the 10 WDS programs located in California’s DACs as of August 2021, one was in the Central Valley. The racial and ethnic demographics of DACs in the Central Valley are as follows: 43% Hispanic or Latino, 35% White, 12% Asian, 7% Black or African American, 2% American Indian and Alaska Native, and 1% Native Hawaiian and Other Pacific Islander (compared to the national averages: 19%, 59%, 6.1%, 14%, 1.3%, and 0.3%) (14).

As of September 2022, ethnic minorities (Hispanic or Latino, Asian, Black or African American, and American Indian or Alaska Native) all had higher risk of COVID-19 cases, hospitalizations, and deaths compared to White, Non-Hispanic persons according to the Centers for Disease Control and Prevention (CDC) (15). Moreover, from 2019–2021 the life expectancy decreased by 5.74 years for Hispanic or Latino, 3.84 years for Black or African American, 3.04 years for Asian, and 1.90 years among White, Non-Hispanic populations due to the COVID-19 pandemic in California (16). Historically, residents of the Central Valley have suffered from a disparity in health care access. Specifically, DACs in this region have access to 1.01 hospitals and medical centers on average per 100 k population while the state averages 2.55 hospitals and medical centers per 100 k population (14, 17). The Central Valley is predicted to experience an 18.7% shortage in primary care physicians by 2025 (18).

HCVT established and supports WDS in disadvantaged and rural communities in California’s Central Valley through partnerships with local public health departments, wastewater municipalities, and analytical laboratory partners. HCVT is an extension of WDS implemented in Davis, California through Healthy Davis Together (HDT) (19, 20) in partnership with the Sewer Coronavirus Alert Network (SCAN) (21). WWTPs were selected from communities with high COVID-19 infection rates, below average vaccination rates (based on fully vaccinated individuals), and from public health department recommendations. The present study describes the initial phase of HCVT in three counties (Merced, Stanislaus, and Yolo). We compare temporal trends between SARS-CoV-2 RNA levels in wastewater settled solids and key health metrics collected in each region, and we report on an inter-laboratory comparison of wastewater settled solids analysis. Correlations amongst WDS and health metrics were analyzed for two surges of COVID-19 infections in the region, the first caused by the Omicron BA.1 variant and the second attributed to the BA.2, BA.4, and BA.5 variants (22). We hypothesized that: (1) correlations between wastewater and health metric data in Central Valley communities would remain strong even with lower access to health resources such as clinical testing and hospitals, (2) changes in testing availability and test-seeking behaviors would lead to changes in case:wastewater ratios observed through time, and (3) hospitalization:wastewater ratios would be more stable through time than case:wastewater ratios.

## 2. Materials and methods

### 2.1. Partner engagement and facility onboarding

**Figure 1:**
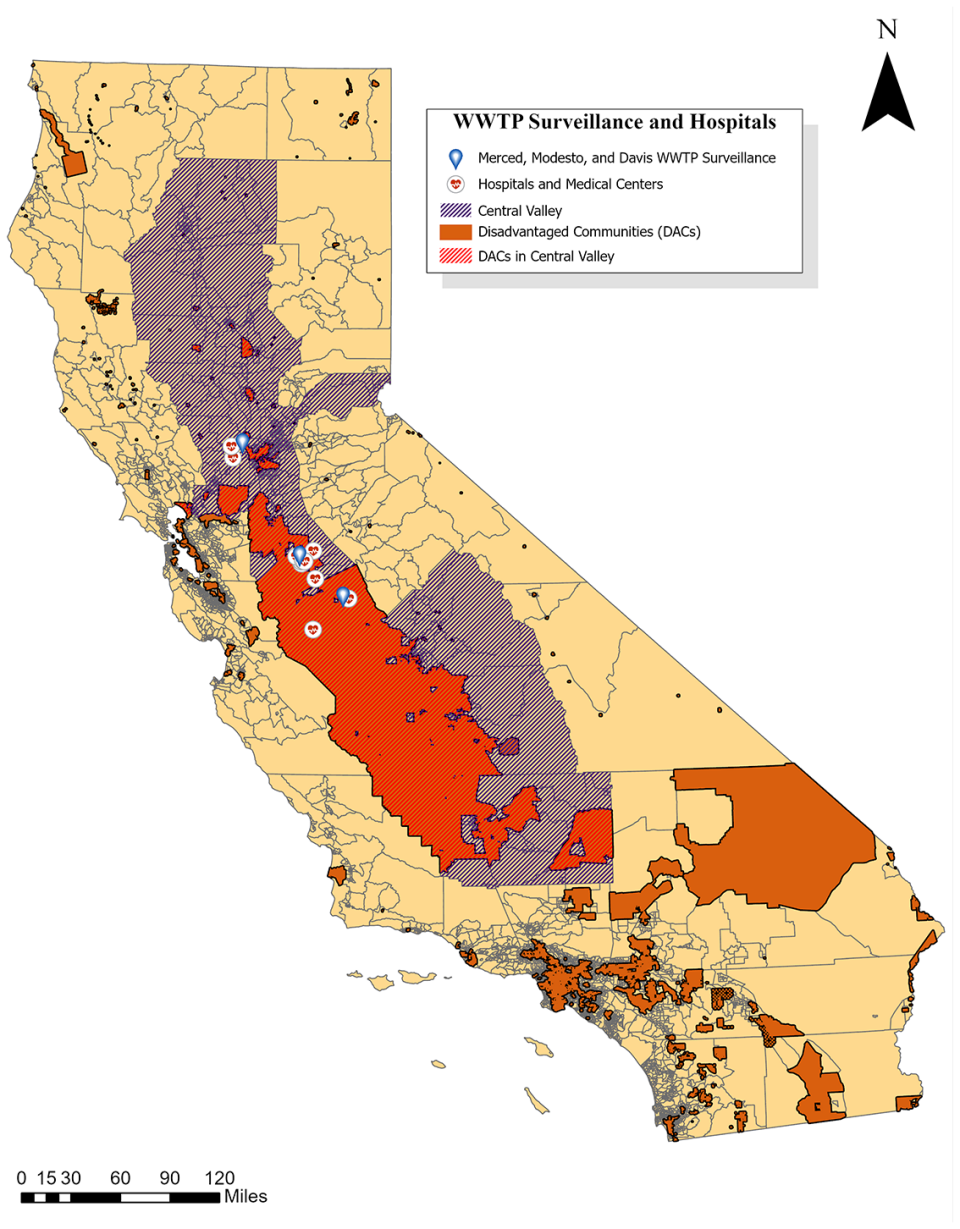
Locations of Merced, Modesto, and Davis WWTPs as well as hospital and medical centers in Merced, Stanislaus, and Yolo Counties compared to disadvantaged communities in California (12, 13, 17, 25, 26).

Merced and Stanislaus Counties were identified as counties of interest due to lower than state average vaccination rates (35% and 39%, respectively, compared to 52% statewide) as of June 2021 (Table 1) (1, 23). Yolo County was selected as a continuation of WDS launched in 2020 through the HDT and SCAN partnership. Vaccination rates by demographic for each county are shown in Supplementary Table S1. Public health departments were consulted to help determine cities for which WDS data would provide value for tracking COVID-19 burden within each county. Figure 1 displays the locations of partner WWTPs in Merced (southernmost location), Modesto (middle location), and Davis (northernmost location) as well as hospital and medical centers in each associated county (1, 17). The Davis WWTP in Yolo County served as an inter-laboratory control for two analytical laboratories used in this study (referred to as Lab 1 and Lab 2) from May through September 2022. Table 2 provides a summary of the sample type and collection frequency, approximate population served (provided by the WWTP), WWTP capacity (MGD), and percentage of industrial input for each treatment plant. Supplementary Table S2 displays city-level data for percent population fully vaccinated, cumulative cases per 100 k population, and total number of hospitals and medical centers (1, 17, 23, 24).

**Table 1:** Comparison of percent population fully vaccinated, cumulative cases per 100k population, and total number of hospitals and medical centers between Merced, Stanislaus, and Yolo Counties to statewide metrics from January 1st, 2021 to June 30th, 2021 (1, 17, 23, 24).

Health metrics in June 2021	Merced County	Stanislaus County	Yolo County	Statewide
Percent population fully vaccinated	35%	39%	55%	52%
Cumulative cases per 100 k population	3,859	3,599	2,267	3,015
Hospitals and medical centers	2	7	2	383

**Table 2:** Characteristics of wastewater treatment plants (WWTPs) and primary clarifier sludge samples collected in this study.

Name of WWTP	Sample Type	Population served	WWTP capacity (MGD)	Industrial input (%)	Samples collected for Lab 1 (date range)	Samples collected for Lab 2 (date range)
Davis WWTP	Primary sludge	70,717	7.5	0	7 days per week (10/20/21–9/29/2022)	4–5 days per week (05/01/22–9/29/2022)
Merced WWTP	Primary sludge	91,000	12	12.5	7 days per week (10/20/21–4/30/2022)	4–5 days per week (05/01/22–9/29/2022)
Modesto WWTP	Primary sludge	230,000	19.1	51.3	7 days per week (10/20/21–4/30/2022)	4–5 days per week (05/01/22–9/29/2022)

### 2.2. Sample collection and handling

This study used wastewater solids for wastewater monitoring. Viral nucleic acids and/or viral particles have been shown to preferentially adsorb to the solids in wastewater in a number of studies. Their concentrations in solids have been shown to be higher by three orders of magnitude compared to wastewater influent (27–32). Wastewater solids represent natural concentrators of viral nucleic-acids, and therefore a useful matrix for carrying out WDS. The study period occurred from October 20, 2021 through September 29, 2022.

#### 2.2.1. Sample processing for lab 1

Samples were handled and processed by commercial partner Lab 1 for Merced and Modesto samples collected prior to May 1, 2022 and for samples collected for Davis throughout the entirety of the study period (Table 2). At each location, grab samples of settled solids processed by Lab 1 were collected 7 days per week directly from the primary clarifier sludge outlet. Methodology followed by Lab 1 for wastewater sample preparation, RNA extraction and droplet digital reverse transcriptase polymerase chain reaction (ddRT-PCR) are described in detail elsewhere (6, 33–35). In short, solids were dewatered using centrifugation and then suspended in a buffer, containing added bovine coronavirus (BCoV) vaccine at 10,000 copies/mL. The solids concentration in that solution was ~75 mg/mL. That solution was then homogenized using bead beating, and then centrifuged, and nucleic acids were extracted from the supernatant. All details for these and additional sample processing steps are precisely provided in other peer-reviewed publications (6) and on protocols.io (33, 34), including the methods for determining the dry weight of solids.

The only modification of sample processing implemented by Lab 1 compared to published methods was a 10-fold dilution of extracts from the city of Modesto implemented from December 30, 2021 to May 1, 2022. Extract dilution was necessary to mitigate inhibition. BCoV was used as a process control and was greater than 10% in all samples. Supplementary Figure S1 shows box-and-whisker plots of the fractional recovery of BCoV. The centerline of the box represents the median value (1.05 for Lab 1). The lower detection limit using these methods is 500–1,000 copies/g dry weight solids for Merced and 5,000–10,000 copies/g dry weight for Modesto after the 1:10 template dilution. The exact value within the range depends on the dry weight of solids which varied.

#### 2.2.2. Sample processing for lab 2

Samples were handled and processed by commercial partner Lab 2 for Merced and Modesto samples collected after May 1, 2022 and for duplicate samples from Davis also collected after May 1, 2022 for an inter-laboratory comparison (Table 2). Lab 2 closely followed the methodology and protocols developed and reported by Lab 1 (33, 36), with sample processing, RNA extraction, and ddRT-PCR methods and modifications detailed in this and the following sections. At each location, grab samples of settled solids for Lab 2 were collected 4 or 5 days per week directly from the primary clarifier sludge outlet in 250-mL HDPE bottles (Environmental Sampling Supply, San Leandro, CA). Reduction in sampling frequency to 4 or 5 days per week from daily allowed for the project to expand sampling to more sites in the region. Chan et al. (37) and Schoen et al. (38) found that a minimum sampling frequency of four or five samples per week was sufficient for acceptable trend analysis. Once collected, samples were immediately stored on ice and transported to Lab 2. If samples could not be immediately transported to the laboratory (i.e., due to weekend sample collection), samples were stored at 5°C on site until laboratory transportation. Samples were processed immediately upon arrival and all laboratory processes were completed within 24 h.

Settled solids samples were homogenized by inverting the HDPE bottle multiple times to mix, and a 50 mL aliquot was transferred to a 50 mL conical tube. Settled solids were dewatered by centrifugation at 24,000 × g for 30 min at 4°C. A stock solution of BCoV (BCoV, Calf-Guard Cattle Vaccine, PBS Animal Health) in DNA/RNA Shield (Zymo Research Corporation, Irvine, CA) was prepared at a concentration of 500,000 genome copies/mL. For samples processed prior to July 29, 2022, four or five aliquots of approximately 75 mg of dewatered solids were transferred into new 50 mL conical tubes, weighed, and an appropriate amount of the BCoV solution was pipetted into each tube to achieve 1 mL DNA/RNA shield per 75 mg dewatered solids. For samples processed from July 29, 2022 onwards, a single 750 mg aliquot of dewatered solids was diluted to the same final ratio of DNA/RNA shield to settled solids mass.

RNA was extracted from dewatered solids using the MagMAX^™^ Microbiome Ultra Nucleic Acid Isolation Kit (Applied Biosystems by Thermo Fisher Scientific) following manufacturer protocols (Pub. No. MAN0018071 Rev. C.0). This protocol deviates from the published methods from Lab 1 by use of KingFisher Flex in place of the Perkin Elmer Chemagic 360. Positive extraction controls (BCoV spike, SARS-CoV-2 genomic RNA: ATCC VR-1986D^™^ and PolyA: Roche 10108626001) and negative extraction controls (nuclease-free water) were included to check for process validity and to ensure no contamination. RNA extraction was immediately followed by PCR inhibitor removal using the Zymo^™^ OneStep-96 PCR Inhibitor Removal Kit (Zymo Research Corporation, Irvine, CA) following manufacturer protocols, with minor modifications. In the sample preparation stage, silicone plates were prepared by centrifuging for 10 min at 2576 g. After samples were added, the plates were spun again for 6 minutes at 2576 g. The RNA extracts were stored on ice for same-day ddRT-PCR reactions and transferred to −80°C for long-term storage. The median fractional recovery of BCoV was 0.96 for Lab 2 (Supplementary Figure S1).

### 2.3. Digital droplet reverse transcriptase PCR (ddRT-PCR)

The following assays were performed to quantify total SARS-CoV-2 concentrations in wastewater samples: N-gene, BCoV, and pepper mild mottle virus (PMMoV). The design and validation of these assays are described by Wolfe et al. (35) and Topol et al. (34). ddRT-PCR protocols implemented by Lab 1 are described in the preceding references. PMMoV is abundant in human fecal matter and its quantified measurements are used to normalize observed SARS-CoV-2 gene target quantitative measurements (6, 39).

The following information describes methods implemented by Lab 2. Primers and probes were purchased from Integrated DNA Technologies (IDT^1^) and positive controls were purchased from American Type Culture Collection (ATCC), Twist Biosciences and IDT. N-gene was run as a triplex assay along with a SARS-CoV-2 S-gene target and an additional variant-specific mutation target (data not included in this analysis). BCoV and PMMoV were run as a duplex assay. ddRT-PCR was performed in 22.5 μL reaction volumes which included 5.5 μL template, 5.5 μL ddPCR One-Step RT-ddPCR Advanced Kit for Probes (1,864,021, Bio-Rad, Hercules, CA), 2.2 μL reverse transcriptase, and 1.1 μL 300 mM dithiothreitol (DTT). Duplex assays included 2.2 μL primer probe mix and 5.5 μL nuclease-free water. Triplex assays included 3.3 μL primer probe mix and 4.4 μL nuclease-free water. The final concentration of primers and probes in the reactions was 900 nM and 250 nM, respectively (35). Reactions were performed in sets of four replicates for samples collected between May 2, 2022 and May 29, 2022, and subsequently performed in sets of five replicates from May 30, 2022 forward.

Droplets were generated using the AutoDG automated droplet generator from Bio-Rad. PCR was performed on the C1000 Touch Thermal Cycler (Bio-Rad): the cycling conditions were reverse transcription at 50°C for 60 min, enzyme activation at 95°C for 10 min, followed by 40 two-step cycles of denaturation at 94°C for 30 s and anneal/extension at 58°C (for SARS-CoV-2, PMMoV, and BCoV targets) for 1 min. This was followed by enzyme deactivation at 98°C for 10 min, droplet stabilization at 4°C for 30 min, and indefinite hold at 4°C. Droplets were analyzed using the QX200 droplet reader from Bio-Rad. Thresholding was performed on QX Manager (Bio-Rad: QX Manager Software Regulatory Edition Version 1.2) (6). Concentrations of assay targets were calculated as copies per gram of dry weight, details of which are described by Wolfe et al. (35). Calculation of the limit of detection for the ddRT-PCR assay implemented by Lab 2 followed protocols recommended by Bio-Rad Laboratories and with further details are included in the supplementary information (Supplementary Table S3) (35, 40).

### 2.4. County health metrics

California county clinical data for Merced, Stanislaus, and Yolo Counties from October 20, 2021 to September 29, 2022 were downloaded from the Official California State Government website which provided the COVID-19 data from the California Health & Human Services Agency (CHHS). The data accessed included fully vaccinated individuals, hospitalization, and county cases. Fully vaccinated data are defined as follows: “2 Pfizer doses > = 17 days apart, 2 Moderna doses > = 24 days apart, 1 dose of J&J, a combination of Pfizer and Moderna doses > = 17 days apart, three or more vaccination records, or only one dose in IRIS labeled as dose number 2” (23).

Hospitalization data used for this analysis included COVID-19 confirmed patients and ICU COVID-19 confirmed patients. The hospitalized COVID-19 confirmed patients are identified as all inpatients, in ICUs and Medical/Surgical units, with laboratory-confirmed COVID-19 results (excluding patients in affiliated clinics, outpatient departments, emergency departments, and overflow locations awaiting an inpatient bed) (41). The ICU COVID-19 confirmed patients are defined as patients in the ICU at the hospital with a laboratory confirmed positive COVID-19 result which includes neonatal intensive care unit (NICU), pediatric intensive care unit (PICU), and adult (41). Hospitalized and ICU patient data are reported based on the county and zip code the hospital is located in, and does not include the county or zip code of residence for each individual patient. To capture as accurately as possible the number of facilities only housing COVID-19 inpatients, the hospital and medical center data used to calculate the number of hospitals and medical centers in each county excludes rehabilitation centers and specialty hospitals. The hospitalization data is dependent upon the number of hospital beds per inpatient facility, and this was not accounted for in the analysis (42).

County case numbers are defined as laboratory-confirmed COVID-19 cases at dates determined by the date of symptom onset, and represent the county of residence for each case (24). Percentage of positive cases in a population were calculated by dividing the number of positive cases from the data by the population count provided by Merced County (population of 287,420), Stanislaus County (population of 562,303), and Yolo County (223,612). The population values in the data collected were taken from the California Department of Finance (DOF) estimates for January 2022 (23, 24, 41). The data for the number of hospitals and medical centers in each county were collected from CHHS and DOF (17). For additional information on how categories were defined, see the data dictionary for individual data sets provided in reference material (23, 24, 41).

### 2.5. Statistical analysis

A 10-day moving average was applied (the mean of the current day and the previous 9 days) to wastewater data to reduce uncertainty and minimize daily fluctuations of the normalized N gene metric. The cases, hospitalization, and ICU in each figure represent the 7-day moving average at the county-level per 100 k population, accessed from the CHHS dataset (17).

It is expected that a patient who is hospitalized will likely be admitted several days after a confirmed COVID-19 diagnosis or the onset of viremia. Therefore, the number of hospitalizations reported on a specific day will lag cases reported and wastewater data. A correlation analysis was performed with the smoothed data to find the lag between wastewater and each of the metrics analyzed (cases, hospitalizations, and ICU). The lag that provided the highest correlation between these metrics and the wastewater data using a grid search was chosen (Supplementary Table S4). Analyses were carried out for the time period of Wave 1, specified for each county, using the R function “cor” to determine the Pearson correlation between county cases, ICU patients, hospitalizations, and wastewater data.

A Pearson correlation coefficient (*r*) and line of best fit (linear regression, *R*^2^) was calculated and analyzed between health metrics (county-level hospitalization, county-level ICU confirmed patients, county-case data, and the California Department of Public Health provided sewershed case data) and WDS data (the normalized N-gene/PMMoV unitless metric and the non-normalized N-gene concentration in gene copies per gram dry weight). The statistical analysis was conducted between seven different time periods: entire sample collection, Wave 1, Peak 1, Wave 2, Peak 2, and Lab 1 and Lab 2 sample collection periods. The start and end dates for each “wave” (Table 3) were defined by visually identifying surges in infections for each city and selecting boundaries for the minimum wastewater concentrations surrounding a given “peak.” Each “peak” date corresponded to the local maximum N/PMMoV from the 10-day moving average. Correlations assessed for waves included data from the start to end dates indicated for each city. Correlations assessed for peaks used data between the start date and peak date within each wave. Rates of full vaccination were also compiled for each analysis period (Table 4).

**Table 3:** Time periods for COVID-19 Central Valley wastewater statistical analysis.

Treatment plant	Analysis period	Start	Peak	End
All	Full Sampling Period	10/20/2021		09/29/2022
Merced	Wave 1	12/19/2021	01/17/2021	03/08/2022
	Wave 2	05/02/2022	07/14/2022	09/29/2022
Modesto	Wave 1	12/28/2021	01/23/2022	02/26/2022
	Wave 2	05/14/2022	07/13/2022	09/29/2022
Davis	Wave 1	12/19/2021	01/09/2022	03/15/2022
	Wave 2	04/27/2022	06/24/2022	09/29/2022

**Table 4:** Percent of the population fully vaccinated across all three counties at the start and end of Waves 1 and 2 with the percent increase in vaccination after each wave (23).

Estimated Wave time periods	Start Wave 1 (11/30/21)	End Wave 1 (03/01/22)	Percent change Wave 1	Start Wave 2 (04/05/22)	End Wave 2 (09/27/22)	Percent change Wave 2
Merced	48%	53%	10%	53%	55%	2.8%
Stanislaus	52%	56%	7.6%	57%	58%	2.2%
Yolo	65%	71%	7.8%	71%	73%	2.2%

### 2.6. Limitations

We note several limitations of this study. First, wastewater surveillance data at the sewershed or city-level was compared to health metrics at the county-level. The wastewater data from one city may not be representative of that county. We selected the largest population centers in each county (Modesto population ~ 230,000, Merced ~ 90,000, and Davis ~ 70,000) to help mitigate this concern. Second, case data and wastewater data are subject to different sources of variability and bias. For instance, case data captures the population with access to healthcare testing and/or infected individual seeking healthcare, while wastewater data is not subject to this bias. Wastewater concentrations are dependent upon fecal shedding of SARS-CoV-2 RNA, and fecal shedding rates are variable per infected individual, while case counts are less impacted by this variability. Third, hospitalization and ICU data is based on individuals admitted and not separated by county of residence. Therefore, individuals seeking care from other counties may result in an overcount of cases in the county with the hospital and an undercount in their county of residence. Our study used the CHHS database, which receives data from the California Hospital Association and reports based on the county of hospitalization (41). Nevertheless, when the patient is at a hospital, they will be shedding SARS-CoV-2 in feces collected by the local treatment plant, which is not necessarily the same WWTP for their residence.

## 3. Results

### 3.1. Trends in health metrics over the study period

**Figure 2:**
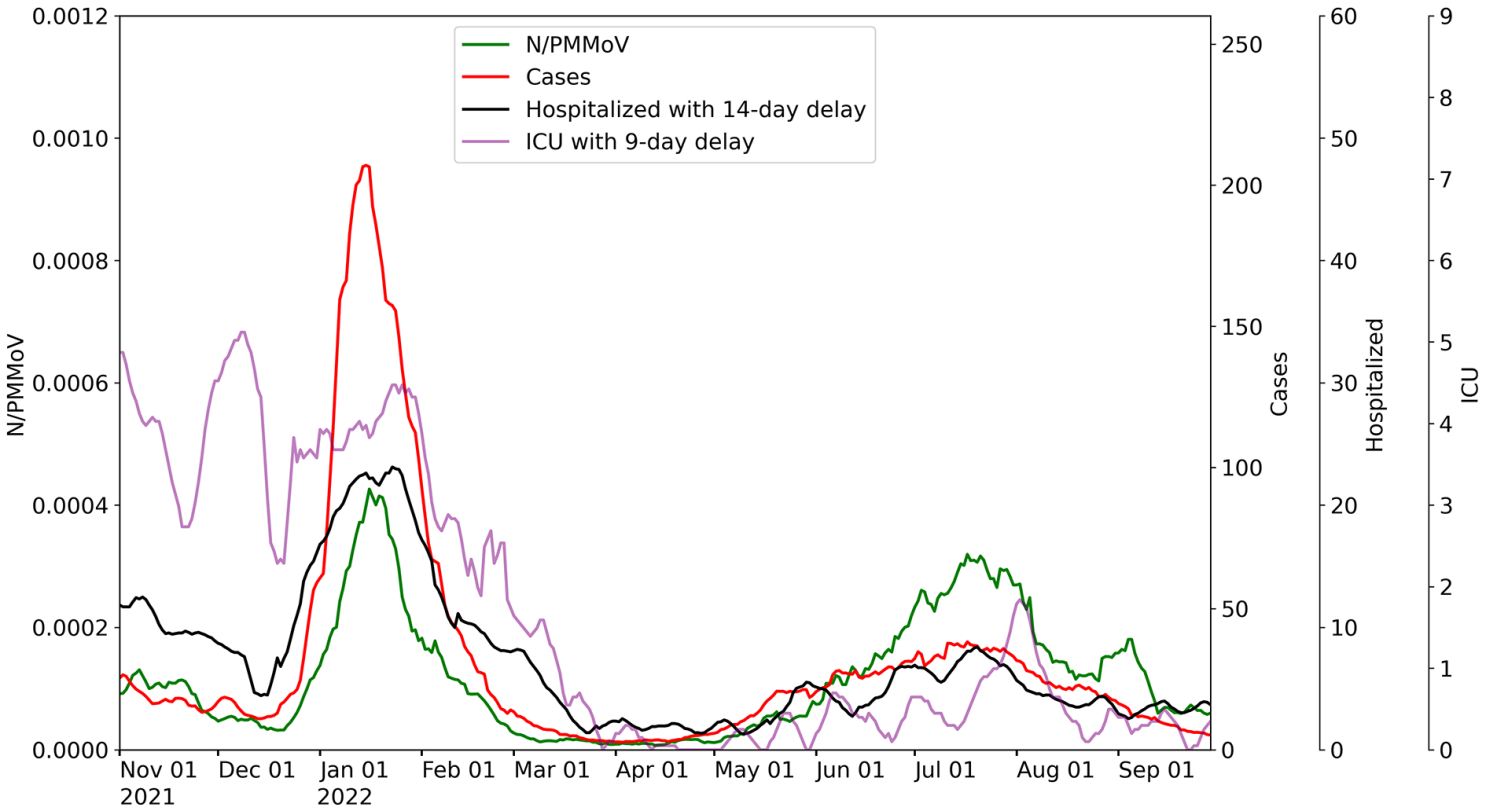
City of Merced wastewater concentrations for 10-day average for N/PMMoV compared to weekly average of Merced County cases per 100 k population, weekly average county hospitalizations with 14-day lag, and weekly average county ICU patients with 9-day lag.

**Figure 3:**
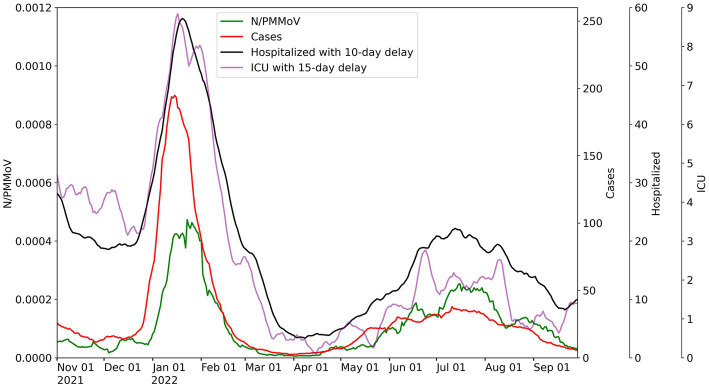
City of Modesto wastewater concentrations for 10-day average for N/PMMoV compared to weekly average of Stanislaus County cases per 100 k population, weekly average county hospitalizations with 10-day lag, and weekly average county ICU patients with 15-day lag.

**Figure 4:**
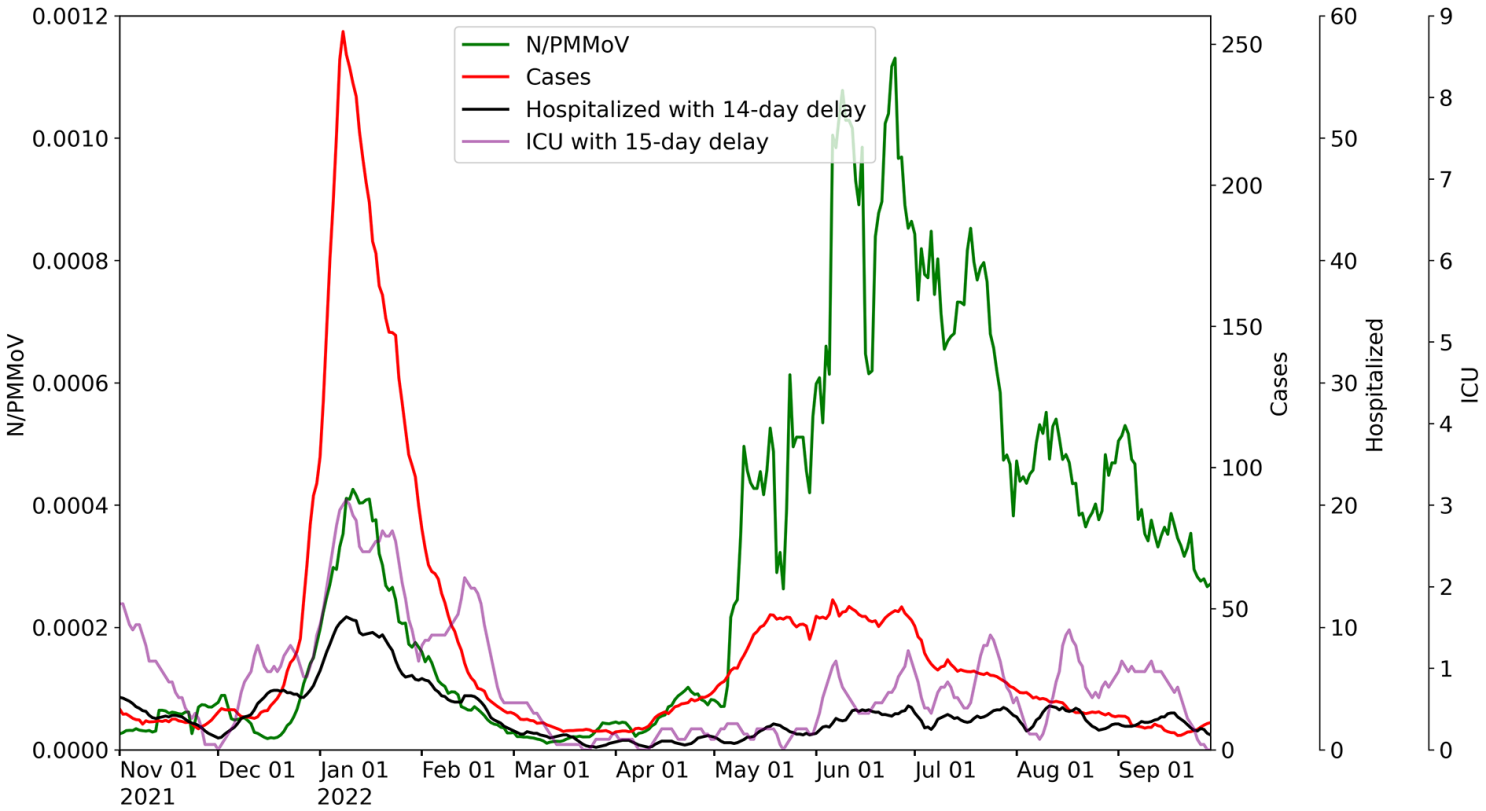
City of Davis wastewater concentrations for 10-day average for N/PMMoV compared to weekly average of Yolo County cases per 100 k population, weekly average county hospitalizations with 14-day lag, and weekly average county ICU patients with 15-day lag.

Merced, Stanislaus, and Yolo County health metric data are co-plotted with 10-day moving averages of N-gene/PMMoV wastewater concentrations from November 1, 2021 to September 29, 2022 for the corresponding WWTP monitored in each county (Figures 2–4). Supplementary Figure S2 includes the Davis data for only Lab 1. Wastewater data collected from each treatment plant captured two distinct waves of infections. The first wave of infections during the study period occurred from approximately December 2021 to March 2022 (referred to herein as Wave 1), and corresponds to a surge in infections predominantly from the Omicron BA.1 variant in the region (43). The second wave of infections during the study period occurred from April 2022 to September 2022 (referred to herein as Wave 2), and corresponds to the surge in infections predominantly from the BA.2, BA.4, and BA.5 variants in the region (43).

Throughout Wave 1, Yolo County exhibited a somewhat greater number of cases, but lower levels in ICU patients and hospitalizations, compared to Merced and Stanislaus Counties. Stanislaus County experienced higher hospitalizations and ICU patients, and similar county case counts, compared to Merced County. The local maxima of N/PMMoV determined by Lab 1 for Wave 1 were similar amongst the three treatment facilities (0.00043 for Merced, 0.00047 for Modesto, and 0.00043 for Davis). In Wave 2, there were much fewer cases, hospitalization, and ICU patients in all three counties. Wastewater levels also declined overall for both Merced and Modesto in Wave 2 compared to Wave 1. However, the local maximum of N/PMMoV for Davis increased for Wave 2 compared to Wave 1.

For visualization of correlations amongst health and wastewater data, hospitalizations and ICU patient counts are shifted in Figures 2–4 by the lag period identified to maximize correlations with wastewater data. For Merced County, hospitalizations exhibited a 14-day lag and a 9-day lag for ICU patients. In Stanislaus County, a 10-day lag in hospitalizations and a 15-day lag for ICU patients was identified compared to the wastewater data. Yolo County exhibited a 14-day lag for hospitalizations, and a 15-day lag for ICU patients compared to the wastewater data. There was no lead or lag identified for wastewater data compared to clinical case data reported by the date of symptom onset.

### 3.2. Inter-laboratory comparison for analysis of settled solids

**Figure 5:**
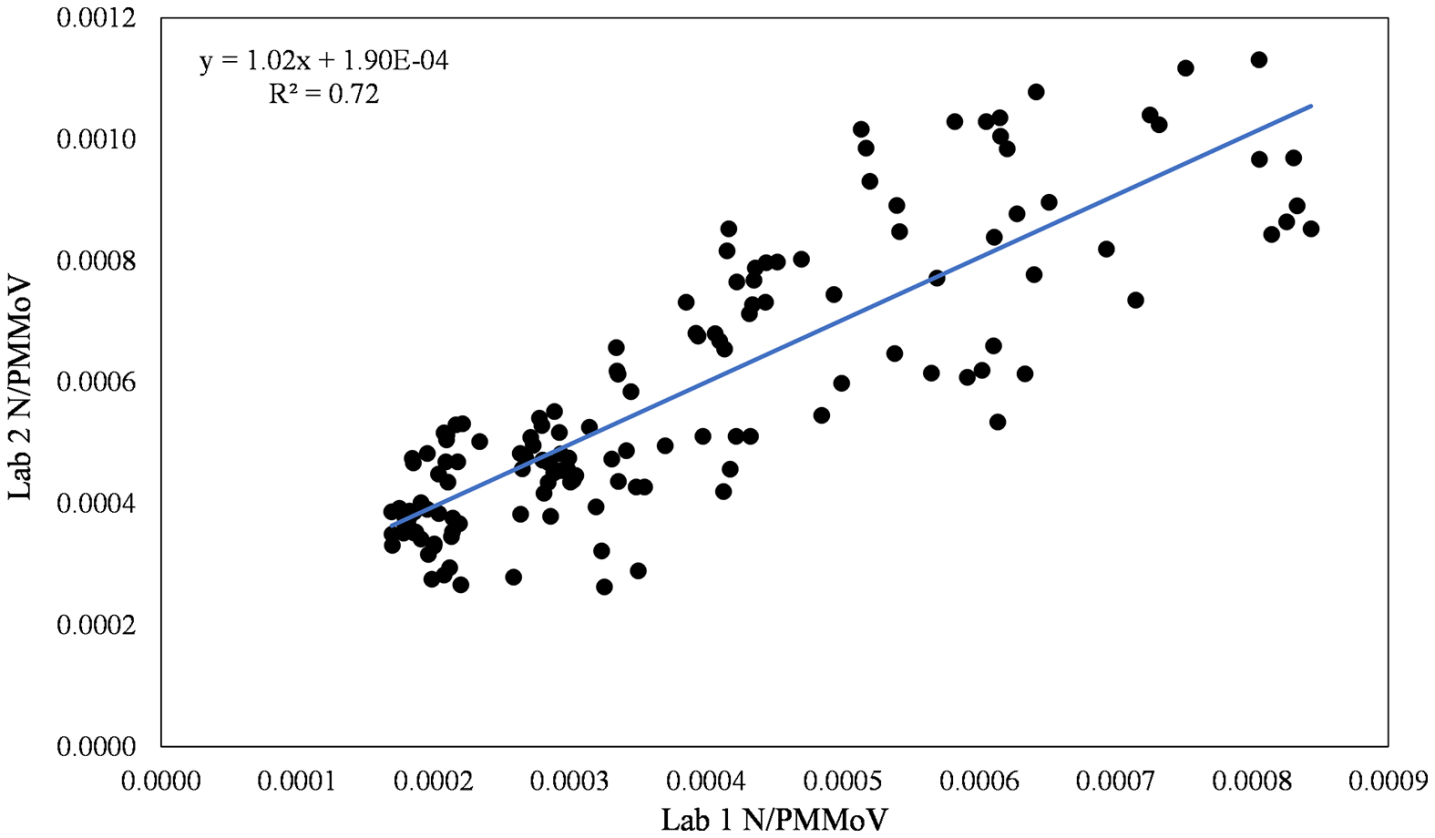
Inter-lab comparison of N/PMMoV between Lab 1 and Lab 2 for the city of Davis.

**Figure 6:**
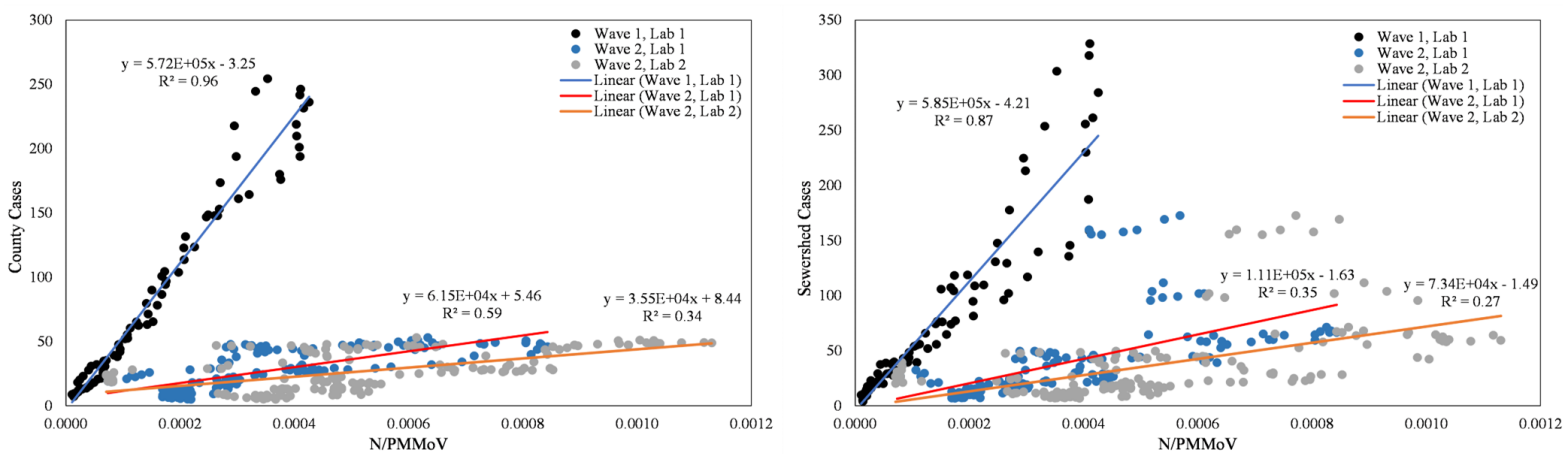
City of Davis N/PMMoV vs. Yolo County cases per 100 k population (left) and city of Davis N/PMMoV vs. sewershed cases per 100 k population (right) between Wave 1 (all Lab 1) and Wave 2 (Lab 1 and Lab 2 separate).

Wastewater settled solids samples collected by Davis during Wave 2 were processed by both Lab 1 and Lab 2 using highly similar analytical methods (modifications of Lab 1 methods that were implemented by Lab 2 are detailed in the methods). N/PMMoV data from the two labs were strongly correlated, with a near 1:1 linear relationship (Figure 5). Lab 2 results yielded somewhat lower concentrations of both N and PMMoV gene copies compared to Lab 1, and similar recovery of BCoV (Supplementary Figures S1, S3-S7). Reporting the normalized statistic (N/PMMoV) helps correct for laboratory variations by using PMMoV as an internal process control (44). The N/PMMoV results determined by Lab 2 were somewhat higher on average than corresponding measurements by Lab 1 (Figure 5). Results indicate overall agreement in trends observed during Wave 2 and demonstrate similar correlations with the health metric data acquired (Figure 6).

**Figure 7:**
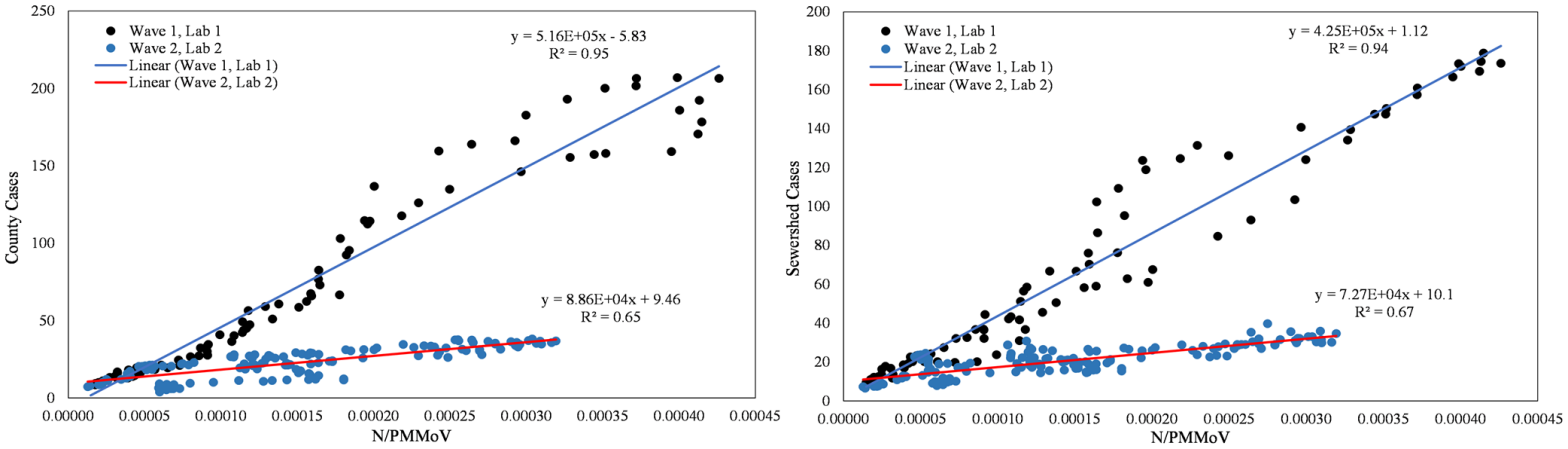
City of Merced N/PMMoV vs. Merced County cases per 100 k population (left) and city of Merced N/PMMoV vs. sewershed cases per 100 k population (right) between Wave 1 (Lab 1) and Wave 2 (Lab 2).

### 3.3. Relationships between wastewater and health metric data for successive surges

**Figure 8:**
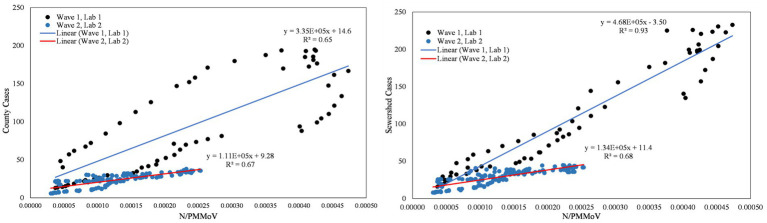
City of Modesto N/PMMoV vs. Stanislaus County cases per 100 k population (left) and city of Modesto N/PMMoV vs. sewershed cases per 100 k population (right) between Wave 1 (Lab 1) and Wave 2 (Lab 2).

**Figure 9:**
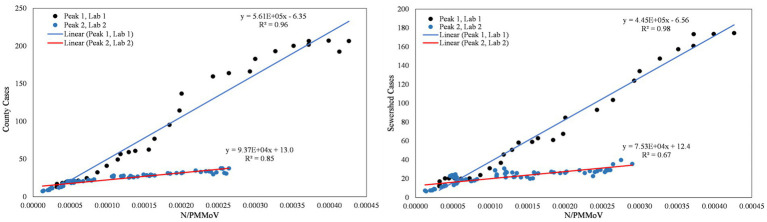
City of Merced N/PMMoV vs. Merced County cases per 100 k population (left) and city of Merced N/PMMoV vs. sewershed cases per 100 k population (right) between Peak 1 (Lab 1) and Peak 2 (Lab 2).

**Figure 10:**
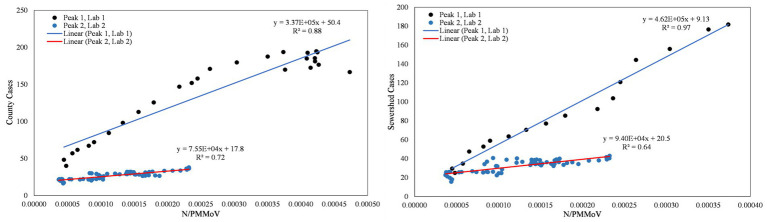
City of Modesto N/PMMoV vs. Stanislaus County cases per 100 k population (left) and city of Modesto N/PMMoV vs. sewershed cases per 100 k population (right) between Peak 1 (Lab 1) and Peak 2 (Lab 2).

**Figure 11:**
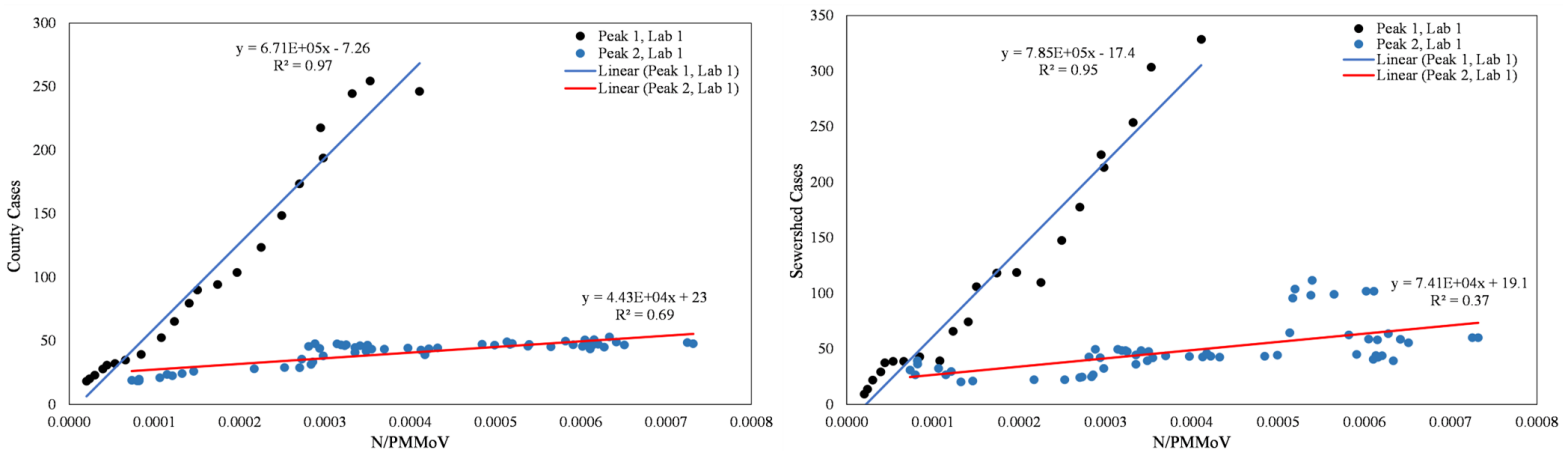
City of Davis N/PMMoV vs. Yolo County cases per 100 k population (left) and city of Davis N/PMMoV vs. sewershed cases per 100 k population (right) between Peak 1 (Lab 1) and Peak 2 (Lab 2).

From visual inspection of Figures 2–4, it is apparent that the relative magnitudes of wastewater results compared to the health metric data changed from Wave 1 to Wave 2. The relationships between the wastewater data and health metric data were thus assessed: (1) over the full study period, (2) for each full wave of infection separately, and (3) using only data on the run-up to each maxima for Wave 1 and Wave 2 (referred to herein as Peak 1 and Peak 2, respectively). Correlations between wastewater data and health metric data were evaluated using linear regressions. Correlations were assessed separately at the county-level and within each WWTP sewershed. The Pearson correlation coefficient (*r*), lag-time delay, and analysis periods for each county for each analysis period are available in Supplementary Table S5. Lines of best fit (coefficient of determination, *R*^2^) between wastewater data and health metric data for each wave of infection are displayed in Figures 6–8. Figures 9–11 show the relationships between N/PMMoV to county and sewershed cases for Peak 1 and 2 (including only data leading up to the maxima of each wave of infection). Correlation plots for wastewater to hospitalization and ICU patient data are in Supplementary Figures S8–S19.

As expected from visual inspection, correlations between health metrics and wastewater data were stronger overall within each wave of infection compared to data assessed over the full study period. The strongest correlations observed were between wastewater data and case data compiled at either the county or sewershed scale. The slopes of the linear relationships for each infection wave were used to determine case:wastewater, hospitalization:wastewater, and ICU:wastewater ratios (Supplementary Table S5, Figures S8–S19). The health metric:wastewater ratios decreased for all counties from Wave 1 to Wave 2 (by 14 to 94%), with the most significant declines generally observed for case:wastewater ratios.

R^2^ values displayed in Figures 6–11 exhibit the percentage variation in y values that is explained by x, signifying the variability between each parameter. Notably for Modesto, lower correlation coefficients (and high variability) within Wave 1 (Figure 8) and high correlation coefficients (low variability) within Peak 1 (Figure 10) signified that wastewater and health metric data increased together, but the metrics were more decoupled on the decline from a peak. Since the slope impacts *R*^2^ values, systematic declines in the coefficient of determination from Wave 1 to Wave 2 are largely statistical artifacts as the slope deviated further from 1:1. The relationships between N/PMMoV to county and sewershed cases are also represented in Supplementary Table S5 through the Pearson correlation coefficient (*r*), the degree of relationship between the two parameters. The Pearson r between wastewater data and health data were generally high, demonstrating strong relationships between health metric and wastewater data (Supplementary Table S5).

## 4. Discussion

Case:wastewater ratios (regression slopes) consistently declined for all three counties from the first surge in infections observed in this study to the second. The average case:wastewater ratio of 4.7 ± 1.4 over the first wave (calculated using county case data) declined to 0.8 ± 0.4 over the second wave. Factors that may lead to systematic declines in case:wastewater ratios include: (1) reduced clinical testing availability and/or participation, (2) replacement of clinical tests by increased use of at-home tests, for which data are not reported to public health officials (45), and (3) changes in the duration or magnitude of fecal shedding (e.g., due to increased rates of vaccination, acquired immunity, new variants, etc.), although little information is available to quantitatively assess this factor (46). Of these factors, we suspect that an increased use of at-home tests and/or changes in test-seeking behavior were especially strong drivers for the declines observed in case:wastewater ratios. While the number of at-home tests conducted in lieu of clinical tests cannot be discerned, discussions with HCVT public health partners affirmed this change in testing behaviors over the study period. More information is needed to fully assess the contribution of fecal shedding dynamics towards changes in case:wastewater ratios through time.

Unlike the case:wastewater ratios, the hospitalization:wastewater ratios and ICU:wastewater ratios (adjusted for lags) remained relatively more stable over the two surges in infections monitored in both Merced and Stanislaus counties. The average hospitalization:wastewater ratio was 0.5 ± 0.3 over the first wave and 0.3 ± 0.4 over the second wave. Hospitalizations are known to be a more reliable indicator of severity of infections and, like wastewater data, are less susceptible to individual choice than test-seeking behaviors. However, hospitalization data within each county is less likely than clinical testing data to be geographically aligned with wastewater data, as patients commonly receive hospital services outside of their residential or workplace jurisdictions. Notwithstanding challenges associated with geographic alignment of wastewater and hospitalization data, the hospitalization:wastewater ratio may be a useful retrospective metric to assess disease severity of an illness, especially when reduced reporting of cases compromises the accuracy of case counts. Wastewater measurements capture information from asymptomatic, mild, and moderate cases, filling data gaps that lead to overestimated hospitalization:case ratios when such cases are counted inaccurately (47).

Three notable differences were observed for Yolo County compared to Merced and Stanislaus Counties. First, a greater number of cases were counted in Yolo County in Wave 1 compared to Merced and Stanislaus Counties. From November 2020 through June 2022, Yolo County and the city of Davis offered community-wide, free clinical testing services through Healthy Yolo Together (HYT) and HDT (19). Participation in each program was high, even for asymptomatic individuals, leading to more comprehensive case counts. Retrospective assessments demonstrated the efficacy of the programs at reducing transmission in the county. The timing of the second infection wave corresponded to the end of the HDT and HYT testing programs (June 30, 2022). The change in programming resulted in dramatic reductions in clinical tests performed in Yolo County.

Second, Yolo County exhibited higher vaccination rates overall. Sixty-five percent of Yolo County was fully vaccinated at the start of the Omicron wave compared to Merced (48%) and Stanislaus (52%) Counties (Table 4). Vaccination rates had increased in each county by 7.8%, 10%, and 7.6% in Yolo, Merced, and Stanislaus Counties, respectively, by the end of the estimated first wave of infections (March 1, 2022).

Third, the hospitalization rates were lower in Yolo County compared to Merced and Stanislaus Counties. On average during the first wave of infections, there were approximately 5 hospitalizations per 100 k population in Yolo County, compared to approximately 15 in Merced County and 41 in Stanislaus County. The approximate average number of ICU admittance per 100 k population for the first wave were more similar amongst the counties, but still lowest in Yolo County (2 in Yolo, 3 in Merced, and 6 in Stanislaus). Hospitalizations and ICU admittance declined in all three counties during Wave 2 compared to Wave 1, with Yolo County maintaining lower rates than Merced and Stanislaus (Supplementary Table S6). Higher vaccination rates in Yolo County for 65+ populations (Supplementary Table S7) may have contributed to the lower ICU admittance and hospitalizations (Supplementary Table S6) observed for Yolo County compared to Merced and Stanislaus Counties. Approximately 93% of the 65+ population in Yolo County was fully vaccinated at the start of the first Omicron wave compared to 78% in Merced, and 83% in Stanislaus Counties.

The results from this study correspond well with other wastewater solids analysis in other locations. Wolfe et al. (6), found strong correlations between SARS-CoV-2 RNA wastewater settled solids concentrations and COVID-19 sewershed cases in eight publicly owned, Northern Californian treatment works from December 2020–March 2021. Wolfe et al. (21) further expanded their analysis to treatment plants in New York and Illinois, and still found strong correlations with COVID-19 cases. The waves of infection captured in our study also have similar timing to the decline in the BA.1 variant in March and emergence of BA.2 in another Californian wastewater solids study (48). Outside of the United States, Hegazy et al. (49) analyzed composite primary clarifier sludge from two treatment plants in Ontario, Canada. They found strong correlations to COVID-19 case rates during the Omicron BA.1 surge (49). Hegazy et al. (49) observed poorer correlations between wastewater data and COVID-19 cases during the Delta wave (July–December 2021), potentially due to higher immunity from vaccinations and prior infection. These findings were similar to the decrease in COVID-19 case correlations observed in Wave 2 (BA.2, BA.4, BA.5) in our study, alongside corresponding increases in vaccinations, boosters, and acquired immunity. Another wastewater solids study that included seven Canadian cities reported changing wastewater to clinical case ratios for different “waves” or dominant variants during the pandemic (50). Similar to our results, Hegazy et al. (49) observed strong correlations with hospitalizations and ICU admissions.

Analysis of wastewater data against health metric data compiled at both the county and city scales offers one strategy to assess population mobility and regional reporting. Wastewater data collected is inherently place-based, while health metrics may be reported in other regions depending on place of residency, place of work, and access to medical centers and hospitals, amongst a myriad of factors. Public health authorities partnered with the HCVT project offered Merced County as one example whereby the closest medical center for residents located in the northern part of the county lies across the county border in Stanislaus County. Further evaluation of wastewater data collected from additional cities across each county is likely to provide insights into disease dynamics across the rural and agricultural communities characteristic of the Central Valley. Integration of wastewater data at regional scales and use of the CalREDIE database (51, 52) for hospitalizations based on county of residence may also offer more representative hospitalization:wastewater ratios when using data from hospitals that serve populations traveling from sewersheds in multiple counties. The California Department of Public Health (CDPH), for instance, assesses population-weighted wastewater data for five public health regions (53) in the state, complementing wastewater data reported at the county and sewershed scales from the CDPH wastewater surveillance network (54).

WDS provides vital public health information to communities by filling gaps in public health data that result from reductions in clinical testing availability, test-seeking behavior, and reporting of test results. WDS data is critical to public health decision-makers in regions where access to and/or utilization of public health resources is inadequate. The HCVT project applied a health equity framework in the selection and implementation of new WDS sites, prioritizing underrepresented regions in California that also exhibited relatively lower vaccination rates, and where higher proportions of the population are identified as disadvantaged. This study demonstrated that WDS still has high correlations with health metric data in areas with lower health care access and reporting. HCVT had open communication on WDS data with public health departments, wastewater treatment plant staff, and city officials from Merced, Stanislaus, and Yolo Counties through weekly email updates, a public website, and bi-weekly meetings throughout the duration of the project. From this communication, public health departments informed local hospitals and skilled nursing facilities of increasing and decreasing wastewater levels. As WDS programs become further integrated into long-term public health decision-making criteria, a critical assessment of WDS using equity metrics should be considered (e.g., evaluating proportional access to WDS data based on racial and ethnic demographics, disadvantaged community status, and access to public health resources). Integration of equity-based WDS program criteria into public health policies will help support initiatives for greater health equity.

## Data availability statement

The datasets analyzed for this study can be found in the Dryad Digital Repository: https://doi.org/10.6071/M3168G.

## Ethics statement

The studies involving human participants were reviewed and approved by University of California, Davis and Stanford University. Written informed consent for participation was not required for this study in accordance with the national legislation and the institutional requirements.

## Author contributions

KK, CN, and HB: contributed to conception and design of the study. KK, CN, GS, MS, RO, MG, AG, JC, BW, AB, MW, and HB: managed and implemented wastewater sample analysis, data collection and sharing, and quality assurance/quality control. KK and GS: contributed to collecting the health metric data for the data analysis of this project and organized the database from each data source. KK, MD-T, JM-L, YG and MN: performed the statistical analysis. CN, HB, MN, AB, MW, MD-T, and JM-L: reviewed each iteration of the manuscript and provided feedback. KK: assembled and wrote much of the first draft of the manuscript and addressed revision comments. KK, CN, HB, RO, GS, MD-T, MG, AG, and JM-L: wrote sections of the manuscript. KK, MD-T, JM-L, RO, and HB: provided tables and figures. RO, AG, and HB: provided equations. All authors contributed to the article and approved the submitted version.

## Funding

The analyses conducted through SCAN were supported by a gift from the CDC-Foundation to AB. Additional research support for HCVT was provided through a philanthropic gift to the University of California, Davis.

## Conflict of interest

MG, AG, and JC are employed by Eurofins Environment Testing US. BW is employed by Verily Life Sciences.

The remaining authors declare that the research was conducted in the absence of any commercial or financial relationships that could be construed as a potential conflict of interest.

## Publisher’s note

All claims expressed in this article are solely those of the authors and do not necessarily represent those of their affiliated organizations, or those of the publisher, the editors and the reviewers. Any product that may be evaluated in this article, or claim that may be made by its manufacturer, is not guaranteed or endorsed by the publisher.

## Footnotes

^1^ https://www.idtdna.com
