# Supplementary material for: Expansion of wastewater-based disease surveillance to improve health equity in California’s Central Valley: sequential shifts in case-to-wastewater and hospitalization-to-wastewater ratios
Source: Front Public Health. 2023 Jun 30;11:1141097. doi: 10.3389/fpubh.2023.1141097 (PMC10348812; doi:10.3389/fpubh.2023.1141097)
Supplement: Supplementary file 1 [file Data_Sheet_1.docx]

**Supplementary Information**

**Table S1.** Percent Population Fully Vaccinated in Merced, Stanislaus, and Yolo Counties per Demographic in June 2021, and percent of demographic per county population [1, 23].

| **County** | **Demographic** | **Percent Fully Vaccinated Per County Population** | **Percent Demographic Per County Population** |
| --- | --- | --- | --- |
| Merced | American Indian or Alaska Native | 0.1% | 0.42% |
|  | Native Hawaiian or Other Pacific Islander | 0.1% | 0.21% |
|  | Black or African American | 0.6% | 2.9% |
|  | Asian | 2.2% | 7.0% |
|  | White | 6.9% | 25% |
|  | Latino | 14% | 61% |
| Stanislaus | American Indian or Alaska Native | 0.20% | 0.48% |
|  | Native Hawaiian or Other Pacific Islander | 0.30% | 0.67% |
|  | Black or African American | 0.70% | 2.6% |
|  | Asian | 2.2% | 6.0% |
|  | Latino | 12% | 48% |
|  | White | 12% | 38% |
| Yolo | American Indian or Alaska Native | 0.20% | 0.44% |
|  | Native Hawaiian or Other Pacific Islander | 0.20% | 0.49% |
|  | Black or African American | 1.0% | 2.7% |
|  | Asian | 7.3% | 14% |
|  | Latino | 13% | 34% |
|  | White | 26% | 38% |

**Table S2.** Comparison of percent population vaccinated, cumulative cases per 100k population and total number of hospitals and medical centers between Merced, Modesto, and Davis cities (by zip code) to statewide metrics from January 1^st^, 2021 to June 30^th^, 2021 [1, 23].

| **Health Metrics in June 2021** | **Merced** | **Modesto** | **Davis** | **Statewide** |
| --- | --- | --- | --- | --- |
| Percent Population Vaccinated | 34% | 40% | 61% | 52% |
| Cumulative Cases per 100k population | 3,405 | 4,060 | 1,058 | 3,015 |
| Hospitals and Medical Centers | 1 | 5 | 1 | 383 |

**Methods: Lab 2 determination of detection limits**

Calculation of the limit of detection (LOD) for the ddRT-PCR assay implemented by Lab 2 followed protocols recommended by Bio-Rad Laboratories (Bio-Rad, 2019). Briefly, the method limit of blank (LoB) was determined by performing four extractions of wastewater with known absence of SARS-CoV-2 N-Gene. Historical wastewater samples absent of SARS-CoV-2 N-Gene were purchased from the Arizona State University Biodesign Center for Environmental Health Engineering. Fifteen replicates for each extraction were analyzed by ddRT-PCR for the target gene for a total of sixty (60) reactions (excluding appropriate QA/QC reactions).

The LoB was calculated by:

$$LoB=Mean_{blank}+1.645*StDev_{blank}$$

Where:

$$Mean_{blank}=calculated mean of limit of blank experiment results$$

$$StDev_{blank}=calculated standard deviation of limit of blank experiment results$$

The calculated LoB was used to estimate the method theoretical limit of detection for calculation of the spike volume of SARS-CoV-2 N-Gene for the limit of detection (LoD) experiment. Four additional wastewater samples with a known spiked volume of SARS-CoV-2 N-Gene were extracted and fifteen replicates of each extraction analyzed by ddPCR for the target gene (total of 60 total reactions excluding appropriate QA/QC reactions).

The LoD was calculated by:

$$LoD=LoB+1.645*StDev_{LoD Experiment}$$

Where:

$$StDev_{LoD Experiment}=calculated standard deviation of LoD experiment results$$

The machine output LoD was calculated to be 0.137 and 0.187 machine output copies/µL reaction for the 75 mg and 750 mg extraction methods, respectively (Table S2). Per CDC NWSS requirements, the calculated limit of detection was converted to units of copies/gram dry weight by:

$$LOD_{gc/g\_dw}=\frac{\left( LOD_{MO} \right)\left( \frac{\left( 20 \mu L volume per reaction well \right)*\left( well\_count \right)}{rna\_template\_vol} \right)\left( \frac{rna\_elution\_vol}{wet\_mass\_g} \right)}{(percent\_solids)}$$

Where:

$$LOD_{gc/g\_dw}=Limit of Detection in gene copies/gram dry weight$$

$$LOD_{MO}=Limit of Detection in machine output copies/\mu L reaction$$

$$well\_count=number of merged wells in ddPCR assay$$

$$rna\_template\_volume=template volume in ddPCR assay across all merged wells$$

$$rna\_elution\_volume=RNA elution volume in ddPCR assay across all merged wells$$

$$wet\_mass\_g=mass of solids across all merged wells for sample$$

$$percent\_solids=calculated percent solids of sample$$

$$20\mu L=volume of extract+master mix per reaction well$$

**Table S3.** Machine output limit of detection and limit of blank for 75 mg and 750 mg extraction methods implemented by Lab 2.

| **Extraction Mass** *(mg)* | **Machine Output Limit of Blank** *(copies/µL reaction)* | **Machine Output Limit of Detection** *(copies/µL reaction)* |
| --- | --- | --- |
| 75 mg | 0.041 | 0.137 |
| 750 mg | 0.056 | 0.187 |

**Table S4.** Time-lag correlation analysis for Merced, Stanislaus, and Yolo Counties between wastewater and county cases, hospitalizations (hospit.), or intensive care unit (ICU). The highest correlation observed is shown in bold and was used to determine the time-lag for each pairing of wastewater with health metric data.

| Lag | Merced/Merced  (12/19/2021 - 03/08/2022) | | | Stanislaus/Modesto (12/28/2021 - 02/26/2022) | | | Yolo/Davis  (12/19/2021 - 03/15/2022) | | |
| --- | --- | --- | --- | --- | --- | --- | --- | --- | --- |
|  | County Cases | Hospit. | ICU | County Cases | Hospit. | ICU | County Cases | Hospit. | ICU |
| 0 | **0.973** | 0.477 | 0.469 | **0.805** | 0.557 | 0.224 | **0.980** | 0.407 | 0.155 |
| 1 | 0.957 | 0.538 | 0.479 | 0.739 | 0.636 | 0.324 | 0.965 | 0.463 | 0.215 |
| 2 | 0.933 | 0.596 | 0.491 | 0.665 | 0.707 | 0.418 | 0.942 | 0.519 | 0.278 |
| 3 | 0.903 | 0.651 | 0.502 | 0.587 | 0.77 | 0.507 | 0.913 | 0.575 | 0.343 |
| 4 | 0.866 | 0.701 | 0.509 | 0.503 | 0.824 | 0.588 | 0.876 | 0.631 | 0.408 |
| 5 | 0.823 | 0.745 | 0.515 | 0.415 | 0.869 | 0.659 | 0.835 | 0.683 | 0.471 |
| 6 | 0.774 | 0.785 | 0.522 | 0.324 | 0.905 | 0.721 | 0.788 | 0.733 | 0.533 |
| 7 | 0.720 | 0.820 | 0.527 | 0.23 | 0.932 | 0.771 | 0.736 | 0.780 | 0.591 |
| 8 | 0.661 | 0.850 | 0.531 | 0.134 | 0.95 | 0.815 | 0.679 | 0.822 | 0.644 |
| 9 | 0.598 | 0.874 | **0.531** | 0.037 | 0.959 | 0.851 | 0.618 | 0.858 | 0.691 |
| 10 | 0.531 | 0.894 | 0.530 | -0.058 | **0.959** | 0.881 | 0.555 | 0.887 | 0.731 |
| 11 | 0.463 | 0.908 | 0.528 | -0.151 | 0.951 | 0.905 | 0.489 | 0.912 | 0.767 |
| 12 | 0.392 | 0.917 | 0.520 | -0.235 | 0.936 | 0.924 | 0.421 | 0.929 | 0.796 |
| 13 | 0.319 | 0.920 | 0.508 | -0.315 | 0.914 | 0.936 | 0.353 | 0.939 | 0.818 |
| 14 | 0.246 | **0.917** | 0.493 | -0.389 | 0.886 | 0.943 | 0.283 | **0.942** | 0.834 |
| 15 | 0.174 | 0.910 | 0.472 | -0.457 | 0.851 | **0.943** | 0.213 | 0.937 | **0.842** |
| 16 | 0.103 | 0.897 | 0.445 | -0.518 | 0.810 | 0.937 | 0.141 | 0.922 | 0.839 |
| 17 | 0.034 | 0.878 | 0.411 | -0.573 | 0.764 | 0.924 | 0.072 | 0.900 | 0.832 |
| 18 | -0.032 | 0.855 | 0.371 | -0.619 | 0.716 | 0.903 | 0.006 | 0.873 | 0.820 |
| 19 | -0.096 | 0.827 | 0.326 | -0.657 | 0.664 | 0.875 | -0.057 | 0.840 | 0.802 |
| 20 | -0.157 | 0.794 | 0.277 | -0.689 | 0.611 | 0.842 | -0.117 | 0.802 | 0.781 |


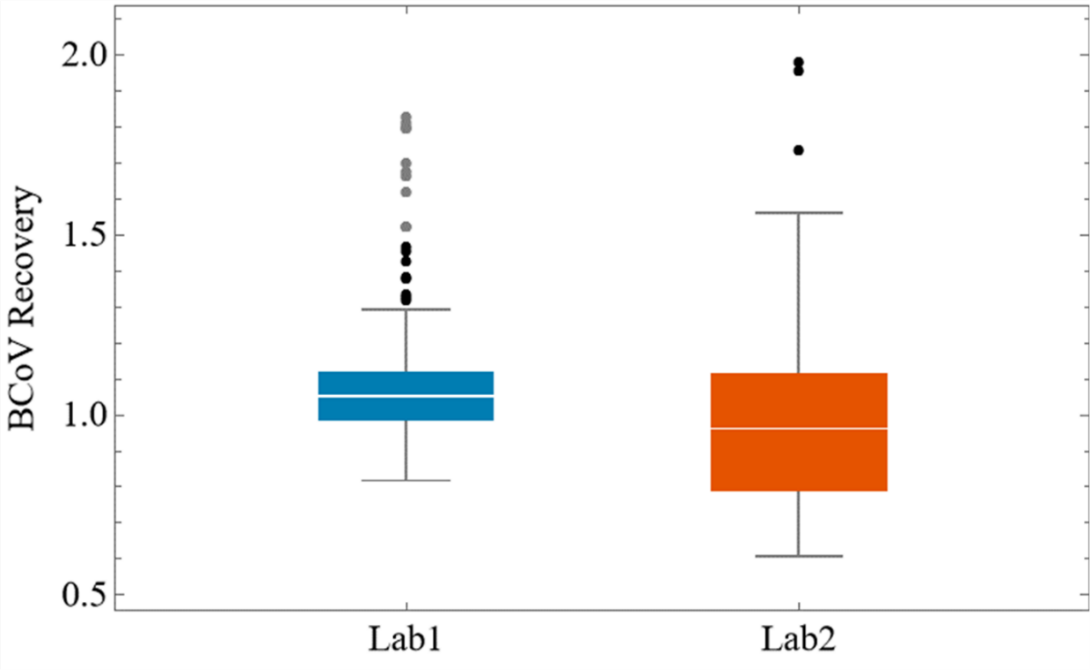


**Figure S1.** Box-and-whisker plots of the fractional recovery of bovine coronavirus (BCoV) determined by Lab 1 and Lab 2. The center line of the box represents the median value, the bounds of the box represent the 25^th^ and 75^th^ percentile results, and whiskers minimum and maximum results without outliers. Outlier criterion was applied to the box-and-whisker plot, and shown as standard outliers (black) and far outliers (grey). Standard outliers are datapoints that are less than or greater than 1.50 x the inter quartile range (IQR), and strong outliers are datapoints that are less than or greater than 3.0 x the IQR.


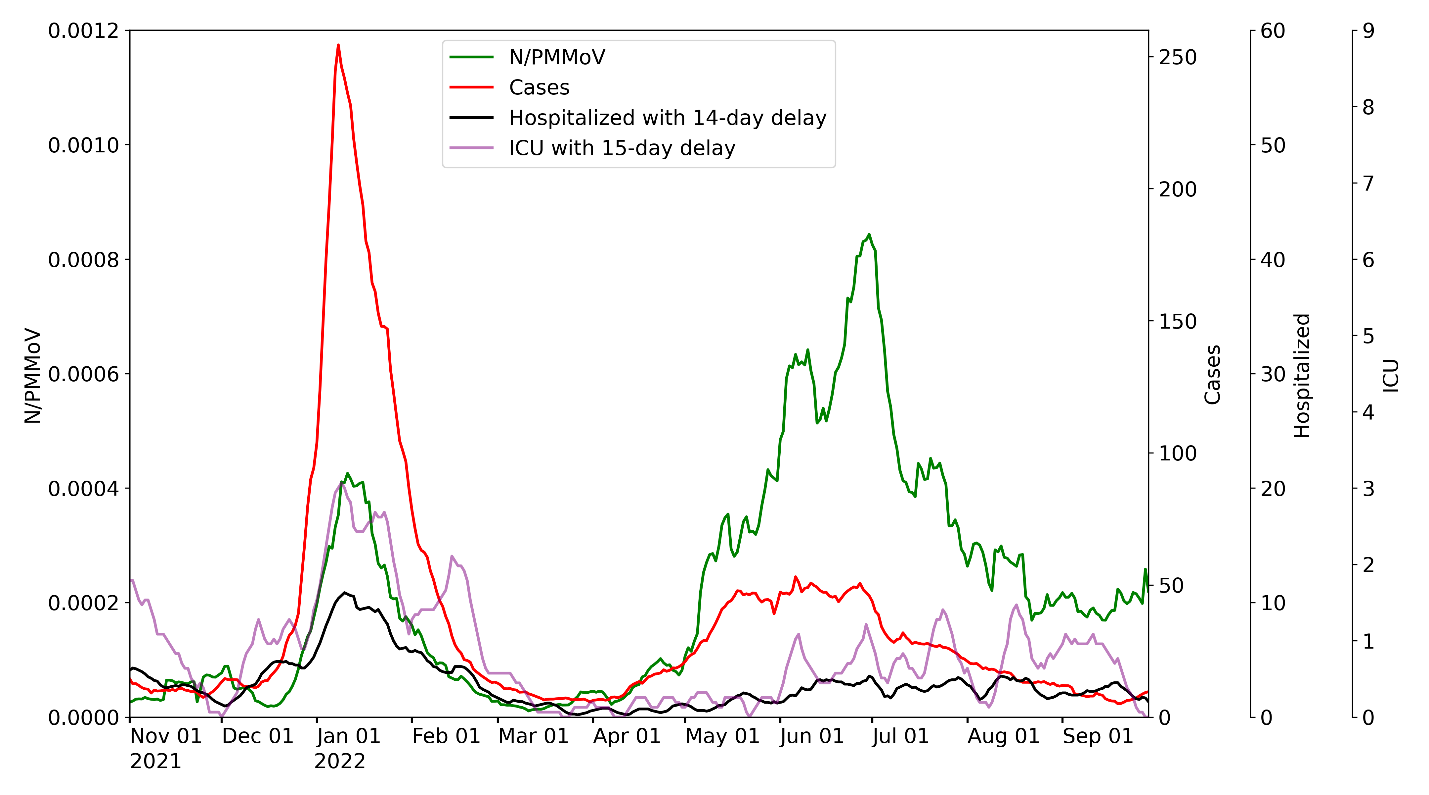


**Figure S2.** City of Davis wastewater concentrations for 10-day average for N/PMMoV (all data was generated by Lab 1) compared to weekly average Yolo County cases per 100k population, weekly average county hospitalizations with 14-day delay, and weekly average county ICU with 15-day delay.


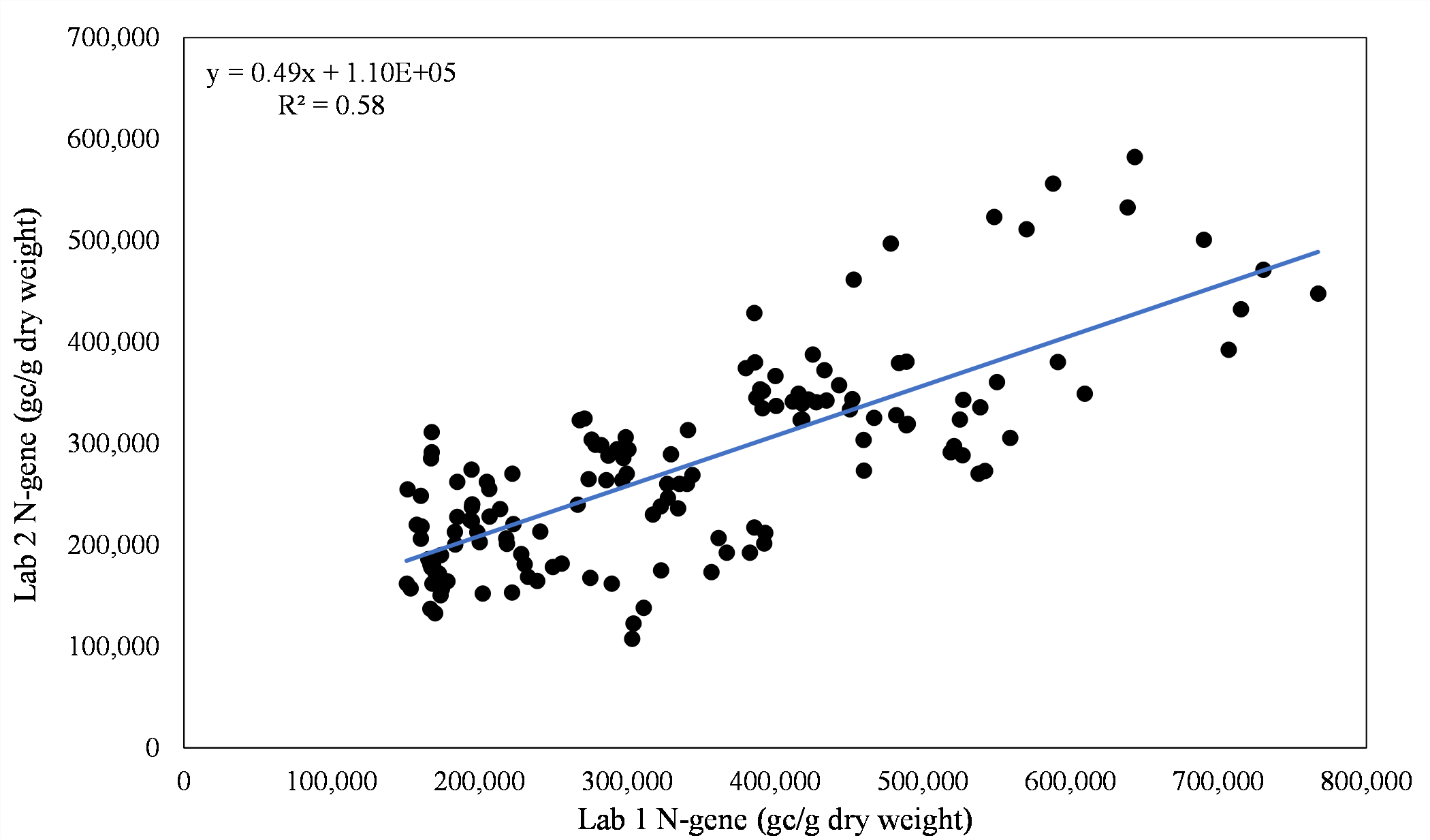


**Figure S3.** Inter-lab comparison of N-gene determined by both Lab 1 and Lab 2 for city of Davis wastewater results.


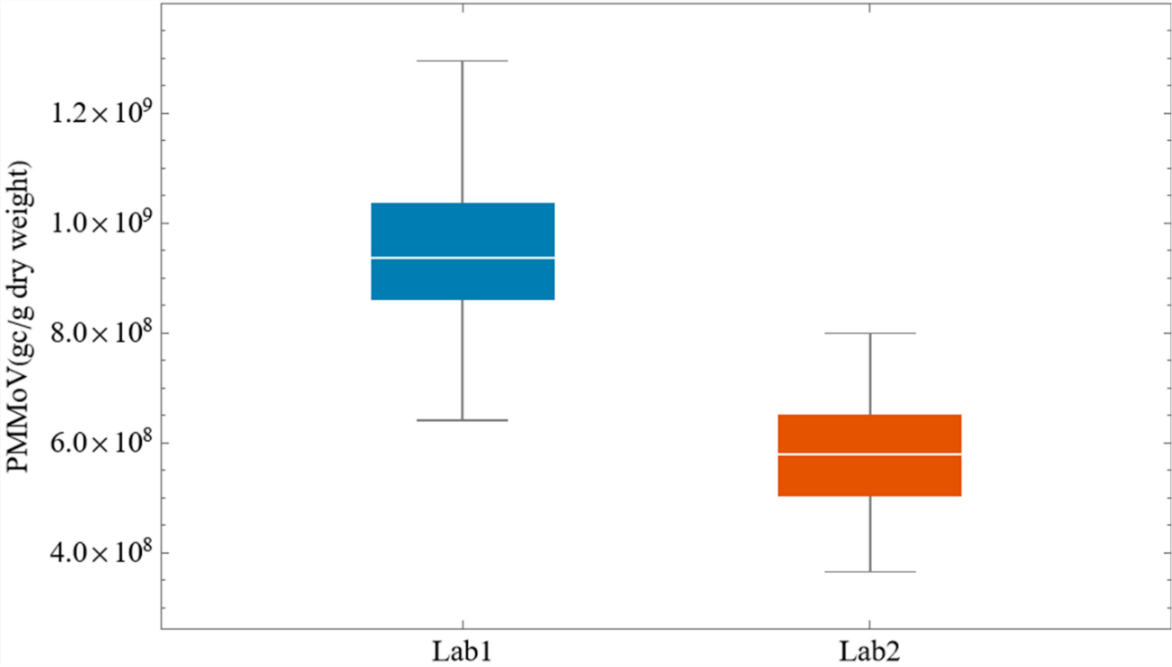


**Figure S4.** Box-and-whisker plots of the concentrations of PMMoV measured by Lab 1 and Lab 2 for city of Davis primary clarifier solids samples. The center line of the box represents the median value, the bounds of the box represent the 25^th^ and 75^th^ percentile results, and whiskers represent the minimum and maximum results. Outlier criterion (1.50 x the inter quartile range) was applied to the box-and-whisker plot and no outliers were determined.


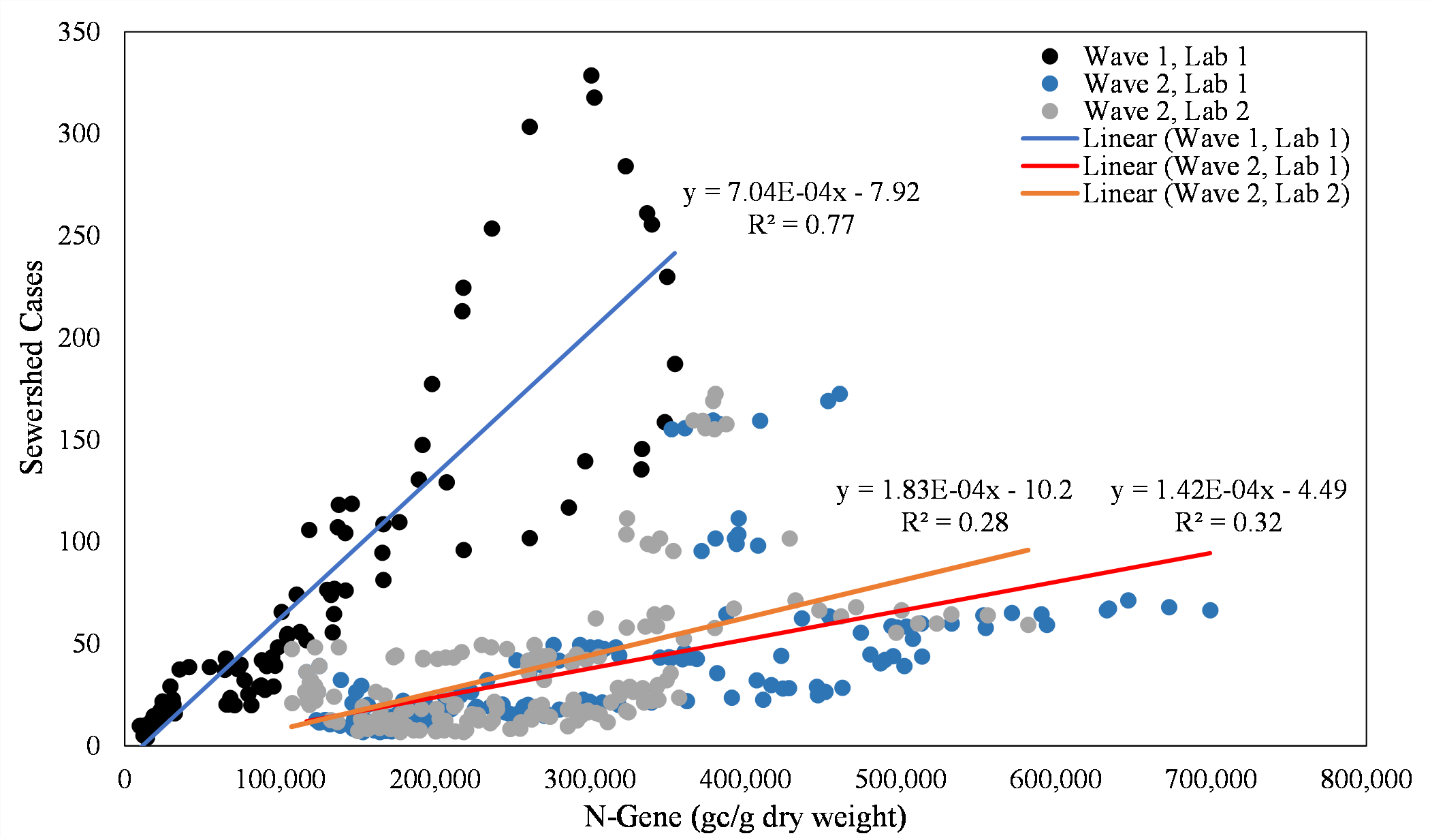


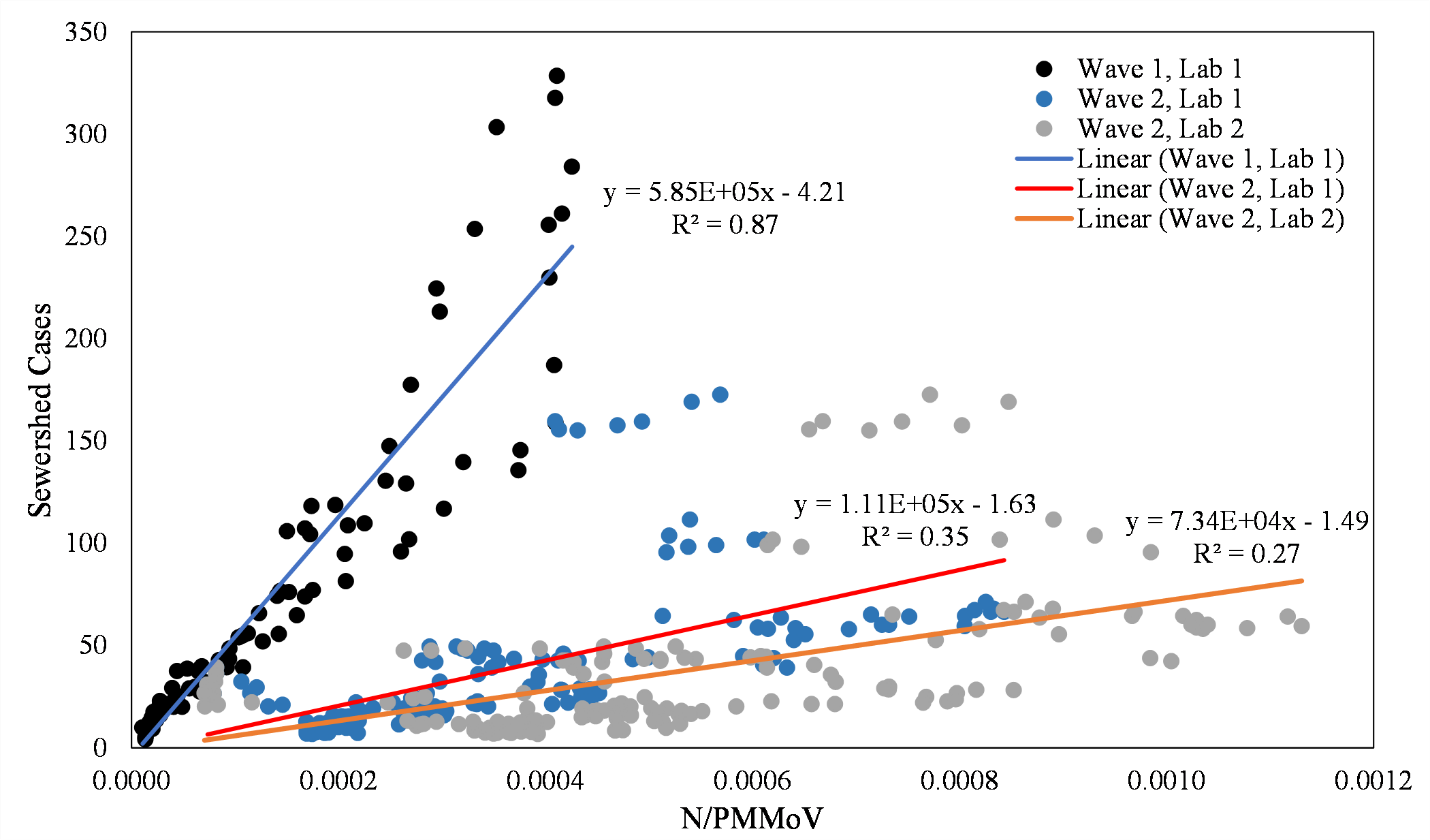


**Figure S5.** City of Davis wastewater results vs. sewershed case data. Sewershed cases were plotted against N-gene concentrations (top plot) and N/PMMoV (bottom plot) for infection Waves 1 and 2.


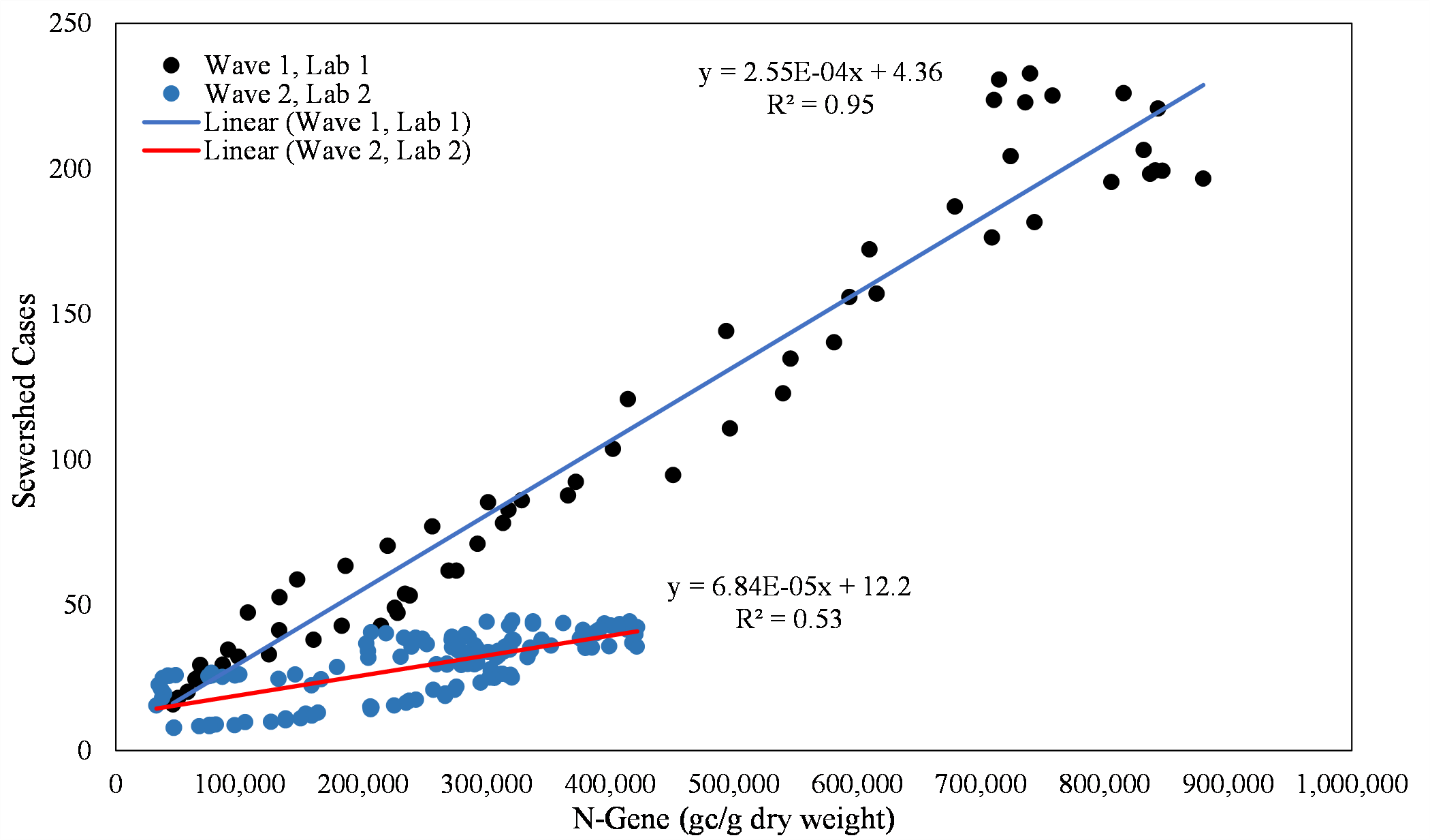


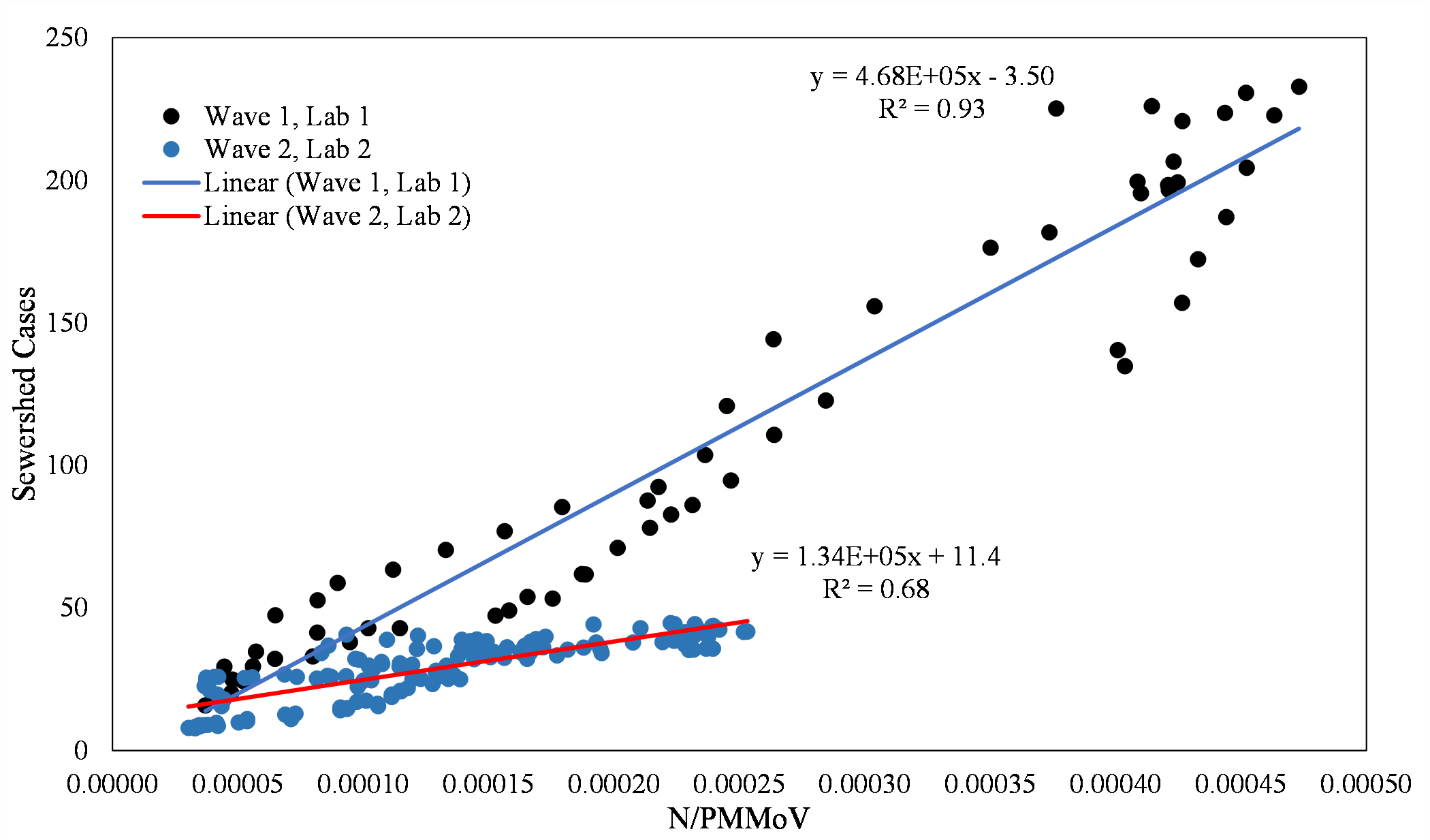


**Figure S6.** City of Modesto wastewater results vs. sewershed case data. Sewershed cases were plotted against N-gene concentrations (top plot) and N/PMMoV (bottom plot) for infection Waves 1 and 2.


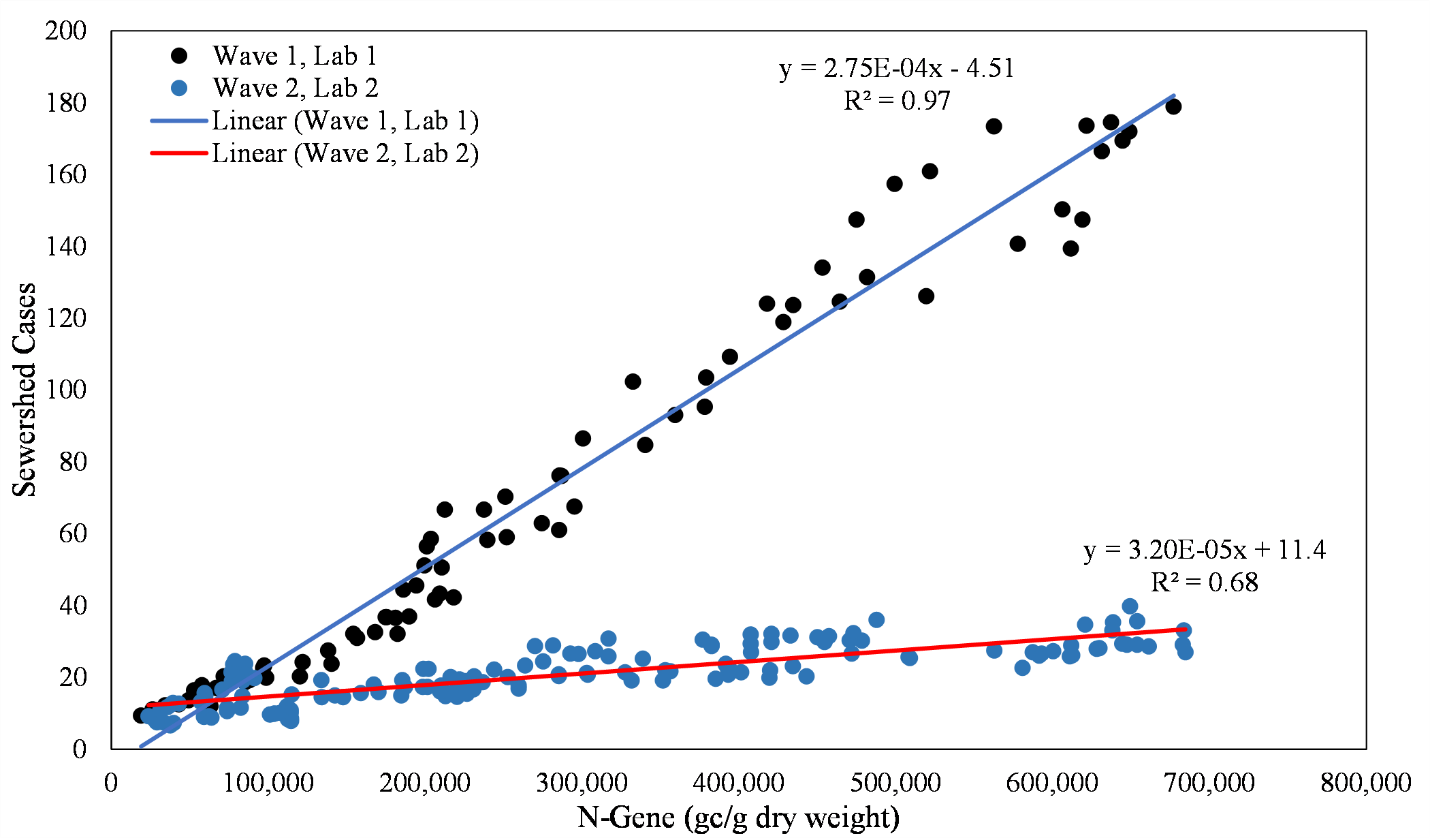


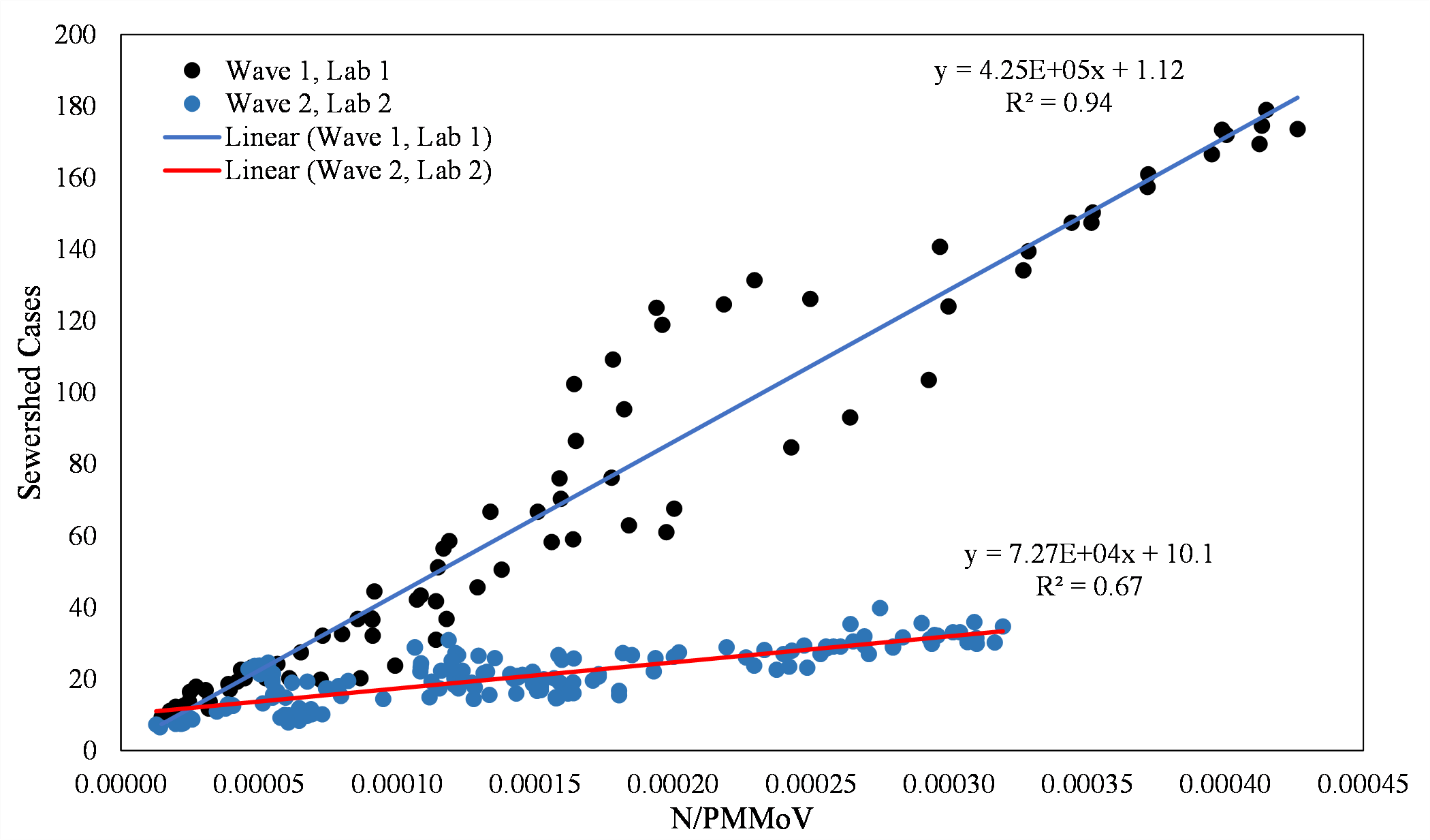


**Figure S7.** City of Merced wastewater results vs. sewershed case data. Sewershed cases were plotted against N-gene concentrations (top plot) and N/PMMoV (bottom plot) for infection Waves 1 and 2.

**Table S5 is available as a separate Excel file.** Pearson correlation coefficient, case:wastewater, hospitalization:wastewater, and ICU:wastewater ratios, confidence intervals, and lag-time for wastewater surveillance data and health metrics between Merced, Stanislaus, and Yolo Counties for seven varying time periods.


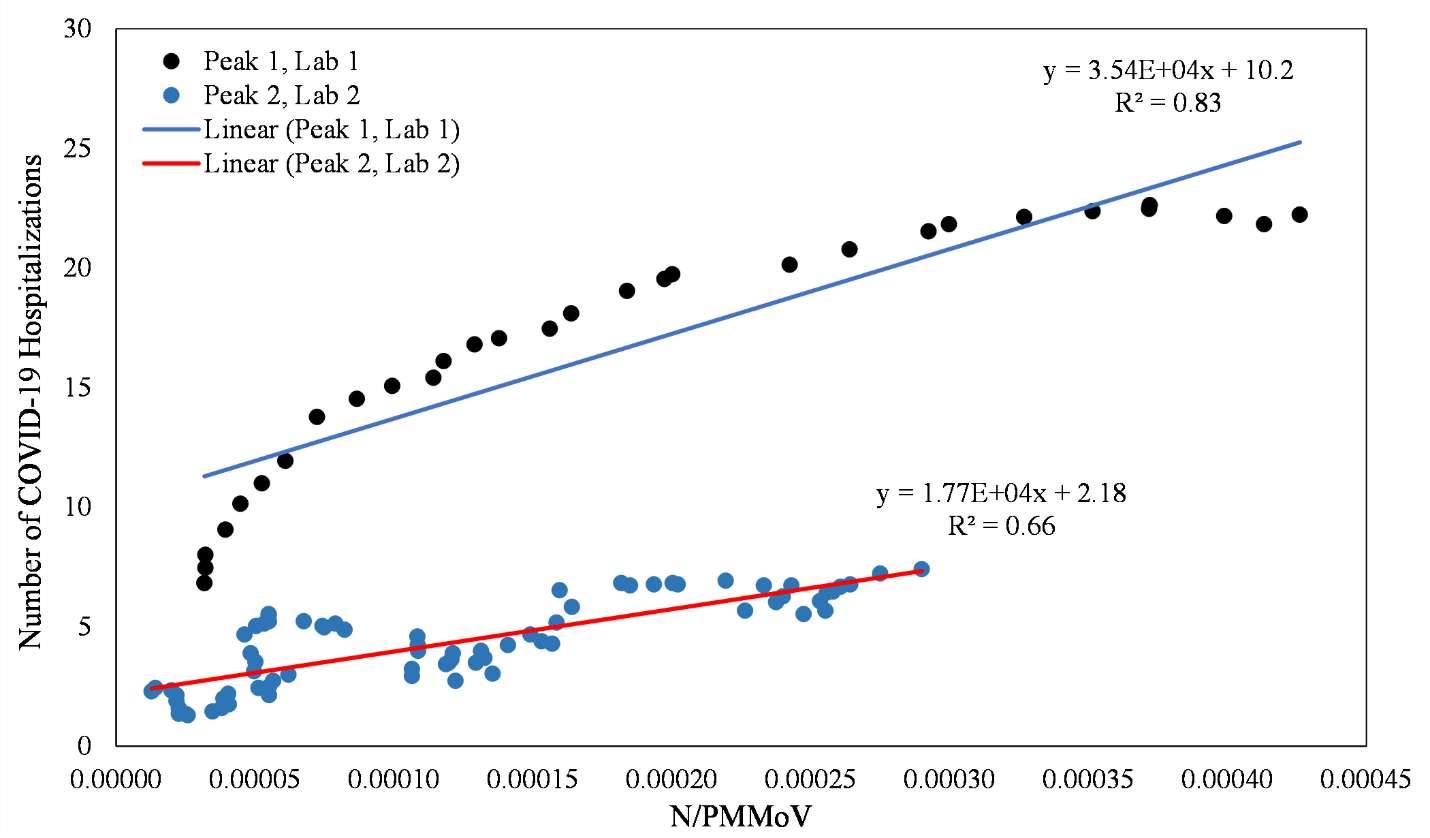


**Figure S8.** Comparison of N/PMMoV for the city of Merced wastewater to Merced County hospitalizations during infection Peak 1 (Lab 1) and Peak 2 (Lab 2).


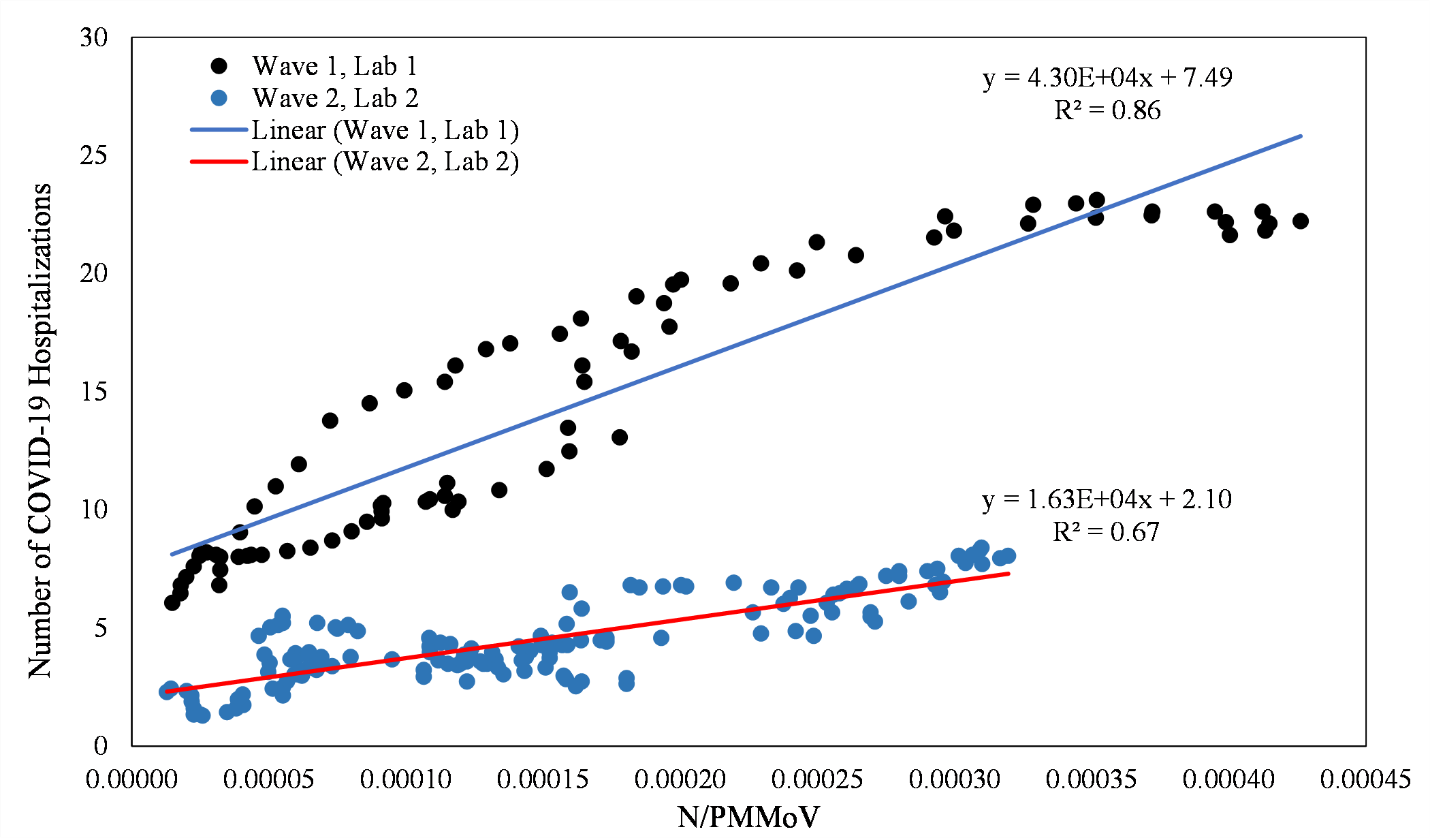


**Figure S9**. Comparison of N/PMMoV for the city of Merced wastewater to Merced County hospitalizations during infection Wave 1 (Lab 1) and Wave 2 (Lab 2).


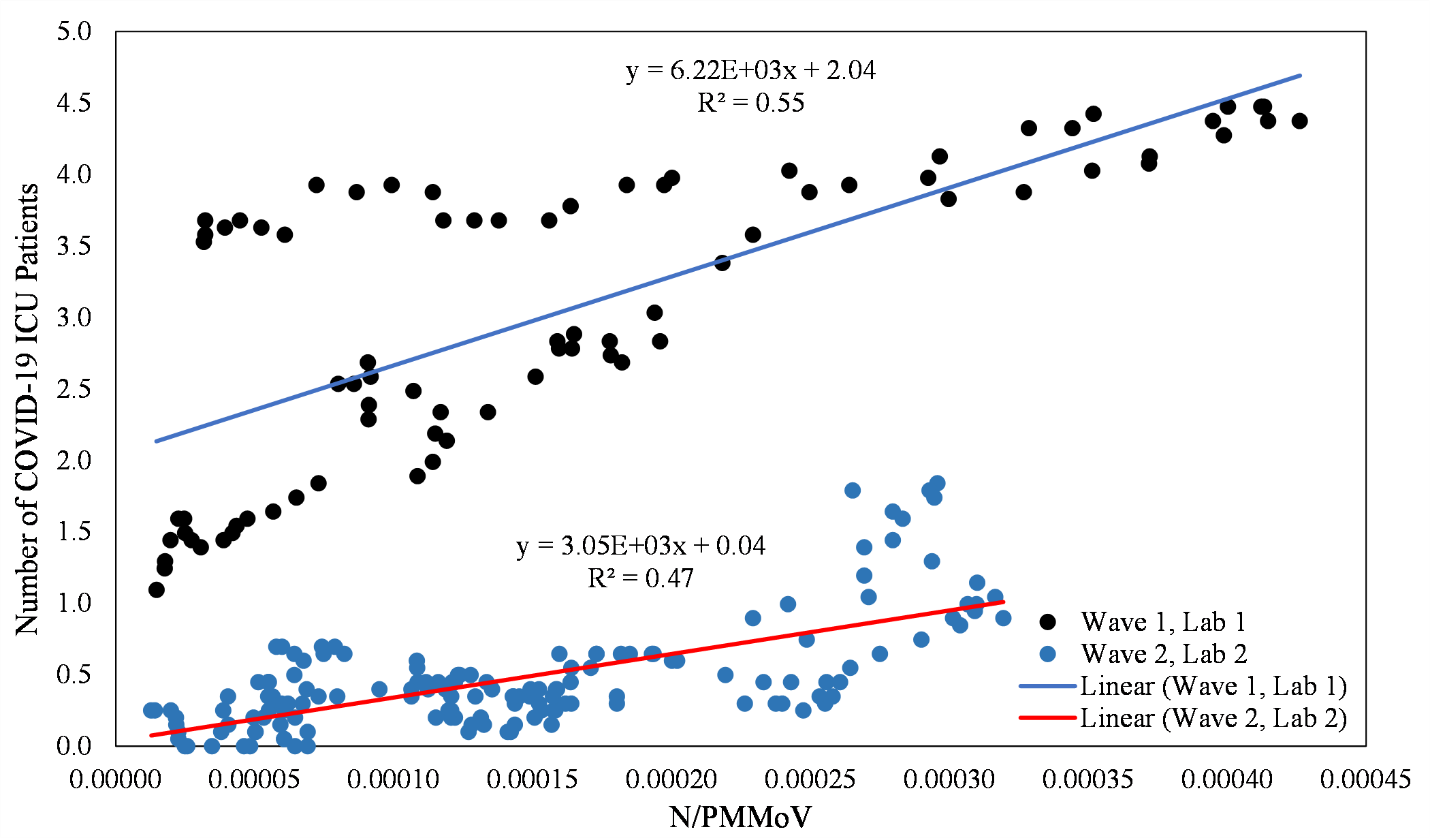


**Figure S10**. Comparison of N/PMMoV for the city of Merced wastewater to Merced County ICU patients during infection Wave 1 (Lab 1) and Wave 2 (Lab 2).


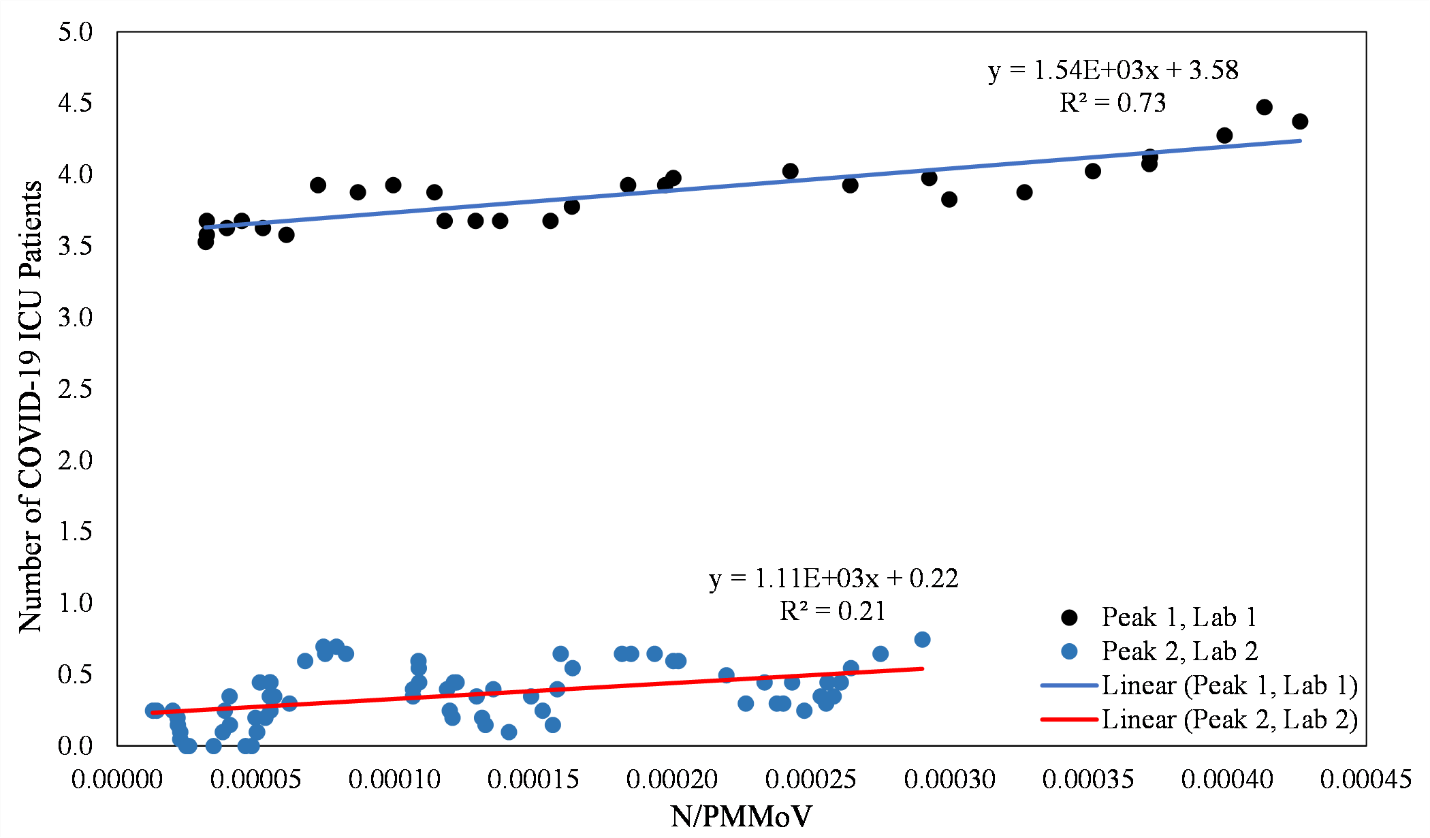


**Figure S11.** Comparison of N/PMMoV for the city of Merced wastewater to Merced County ICU patients during infection Peak 1 (Lab 1) and Peak 2 (Lab 2).


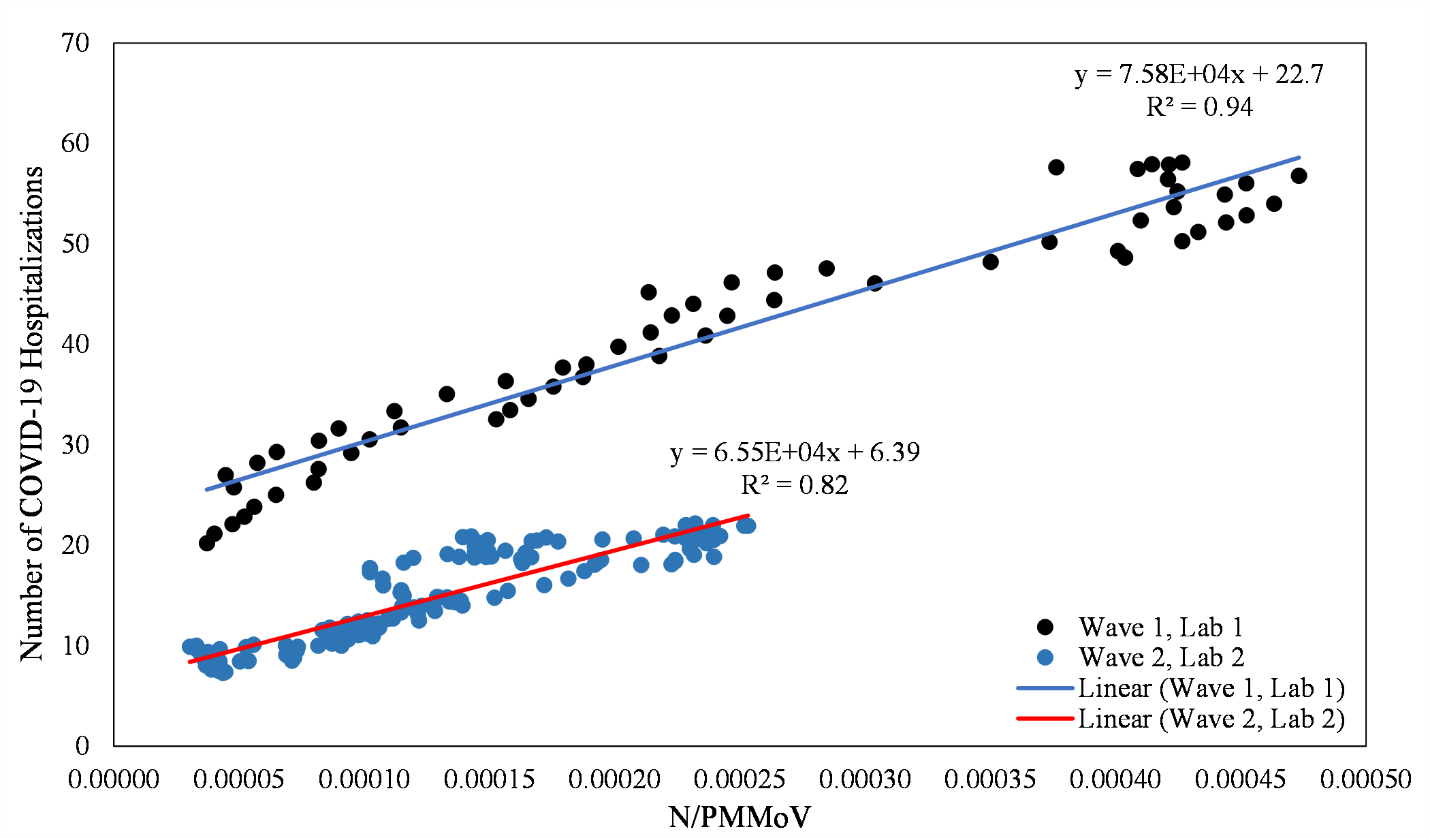


**Figure S12.** Comparison of N/PMMoV for the city of Modesto wastewater to Stanislaus County hospitalizations during infection Wave 1 (Lab 1) and Wave 2 (Lab 2).


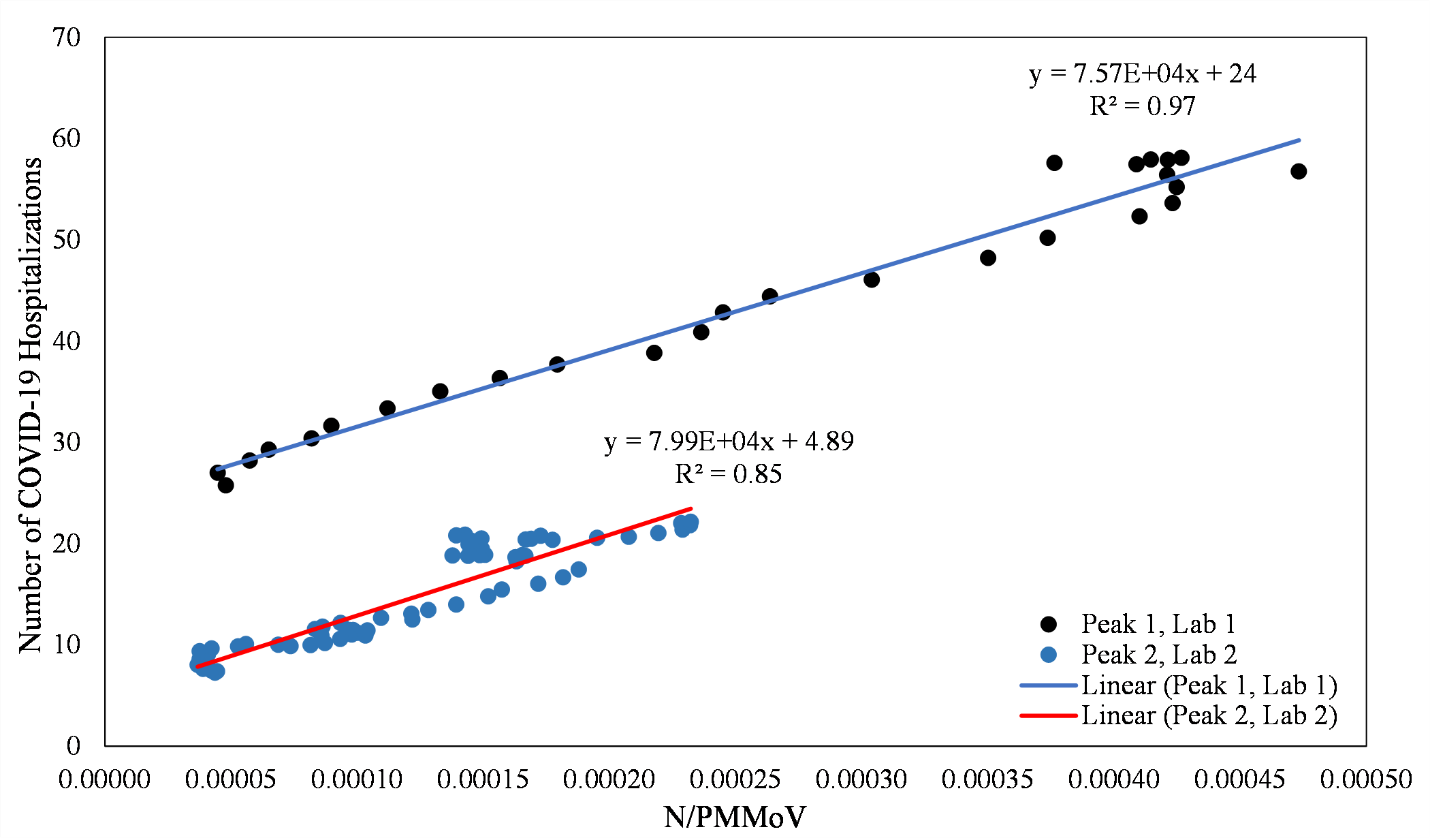


**Figure S13.** Comparison of N/PMMoV for the city of Modesto wastewater to Stanislaus County hospitalizations during infection Peak 1 (Lab 1) and Peak 2 (Lab 2).


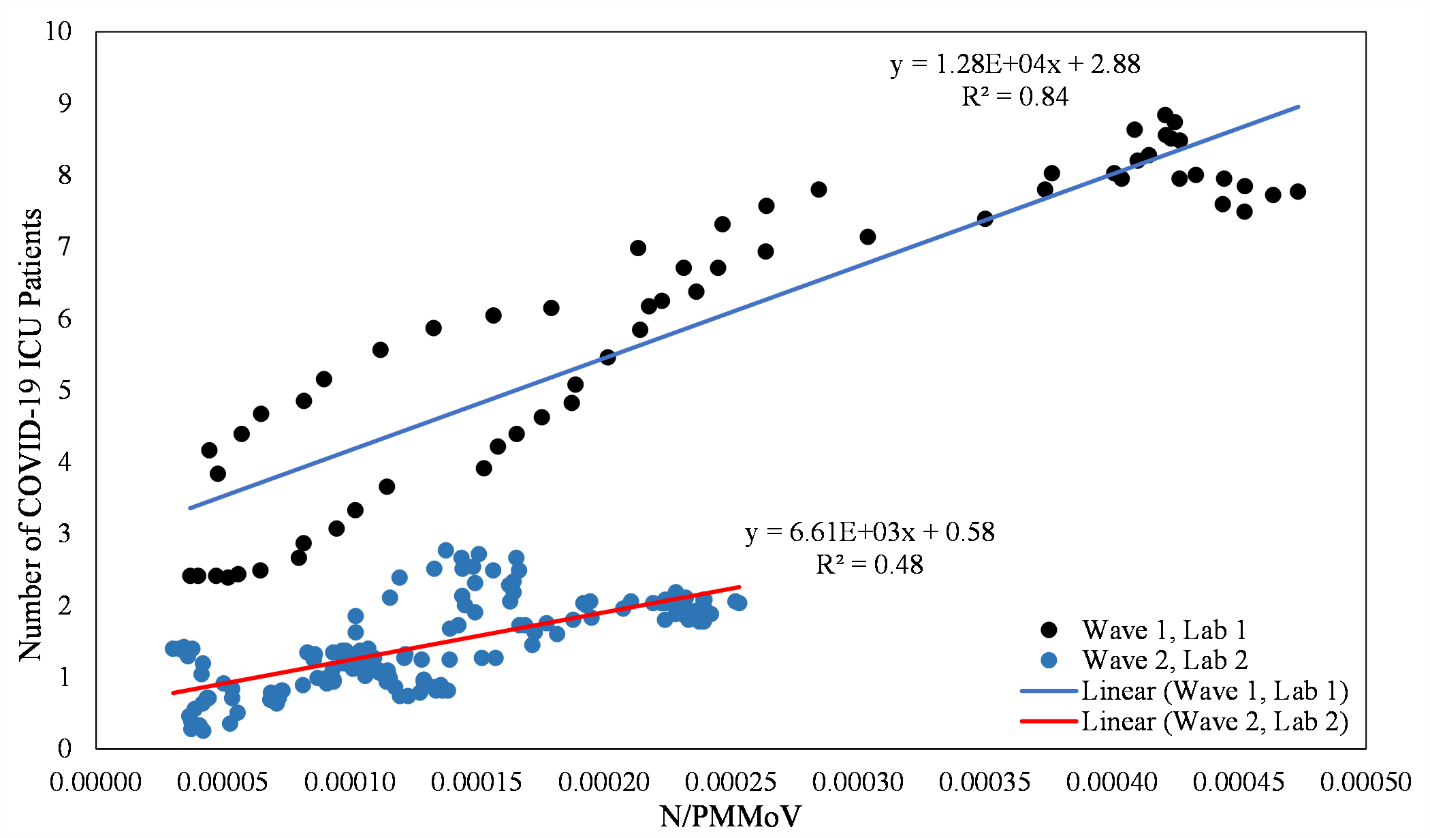


**Figure S14.** Comparison of N/PMMoV for the city of Modesto wastewater to Stanislaus County ICU patients during infection Wave 1 (Lab 1) and Wave 2 (Lab 2).


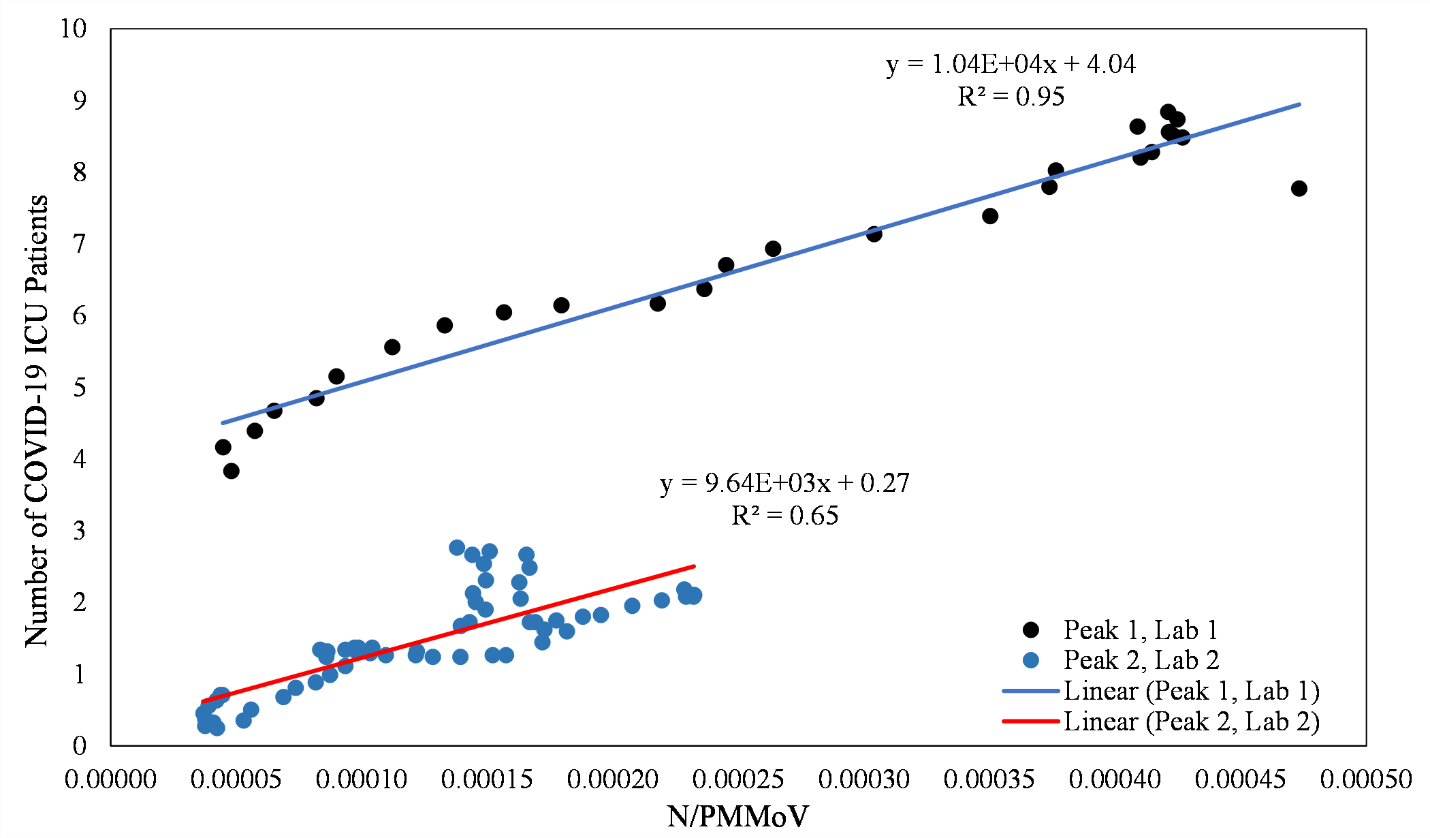


**Figure S15.** Comparison of N/PMMoV for the city of Modesto wastewater to Stanislaus County ICU patients during infection Peak 1 (Lab 1) and Peak 2 (Lab 2).


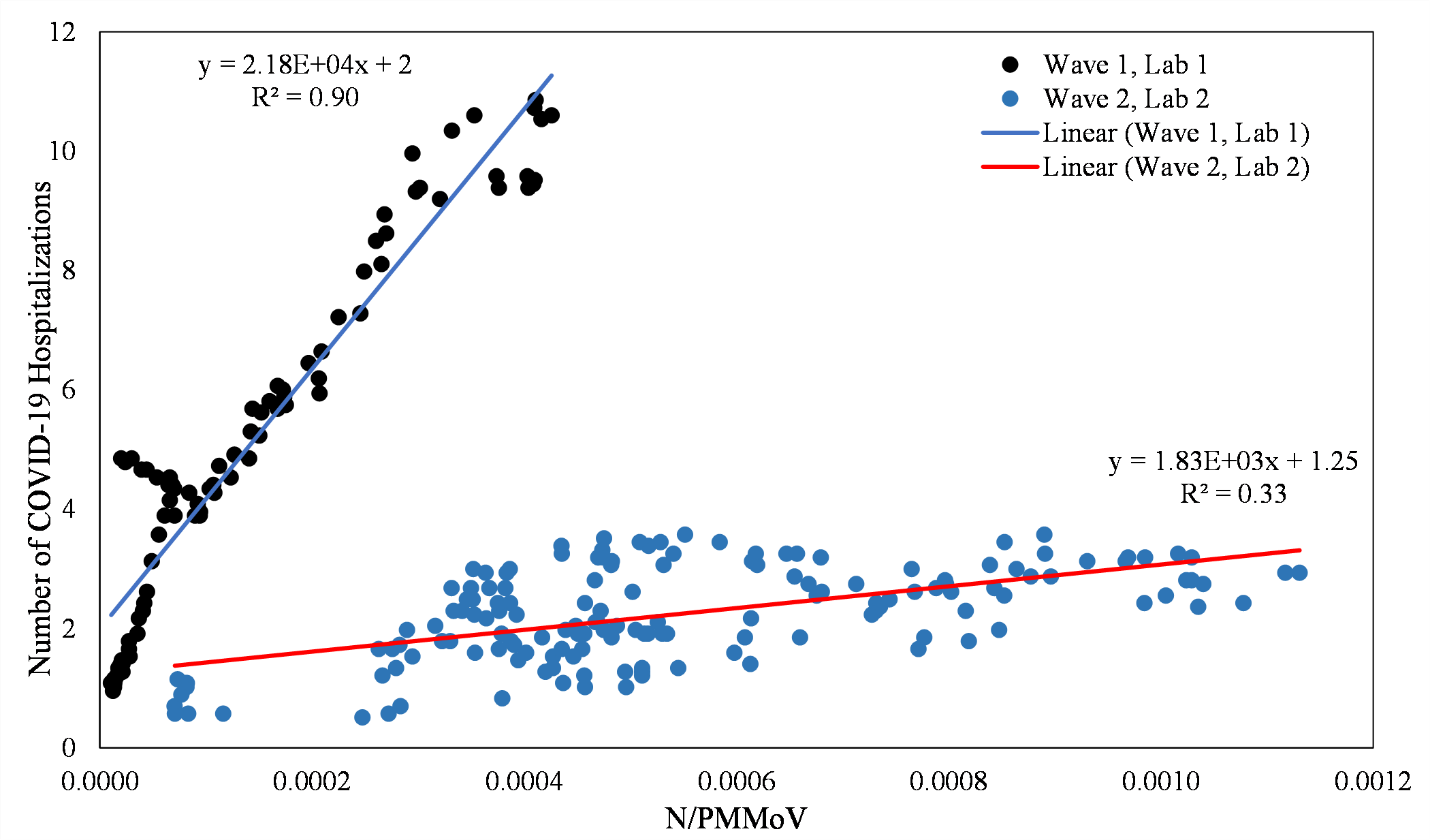


**Figure S16.** Comparison of N/PMMoV for the city of Davis wastewater to Yolo County hospitalizations during infection Wave 1 (Lab 1) and Wave 2 (Lab 2).


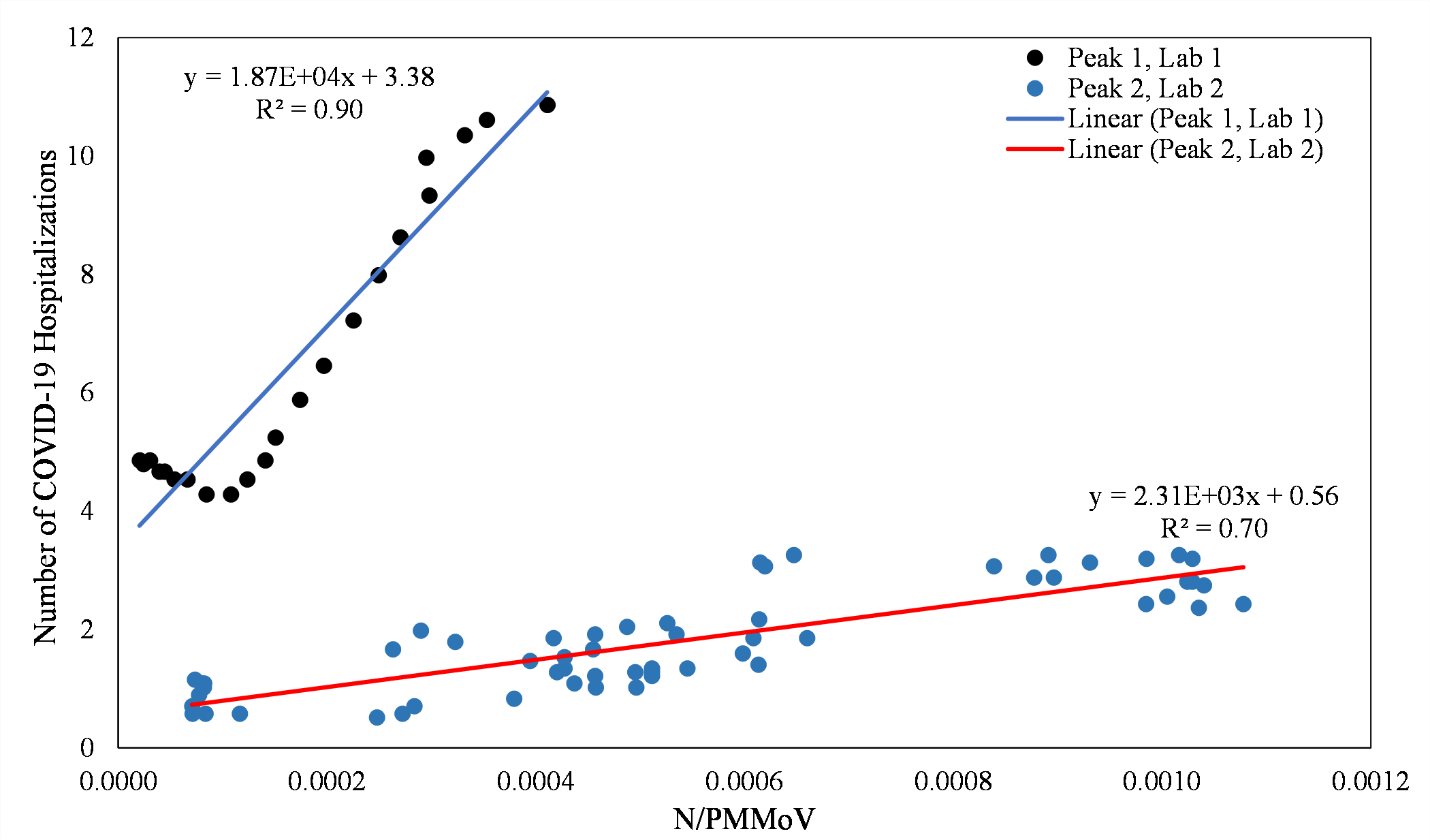


**Figure S17.** Comparison of N/PMMoV for the city of Davis wastewater to Yolo County hospitalizations during infection Peak 1 (Lab 1) and Peak 2 (Lab 2).


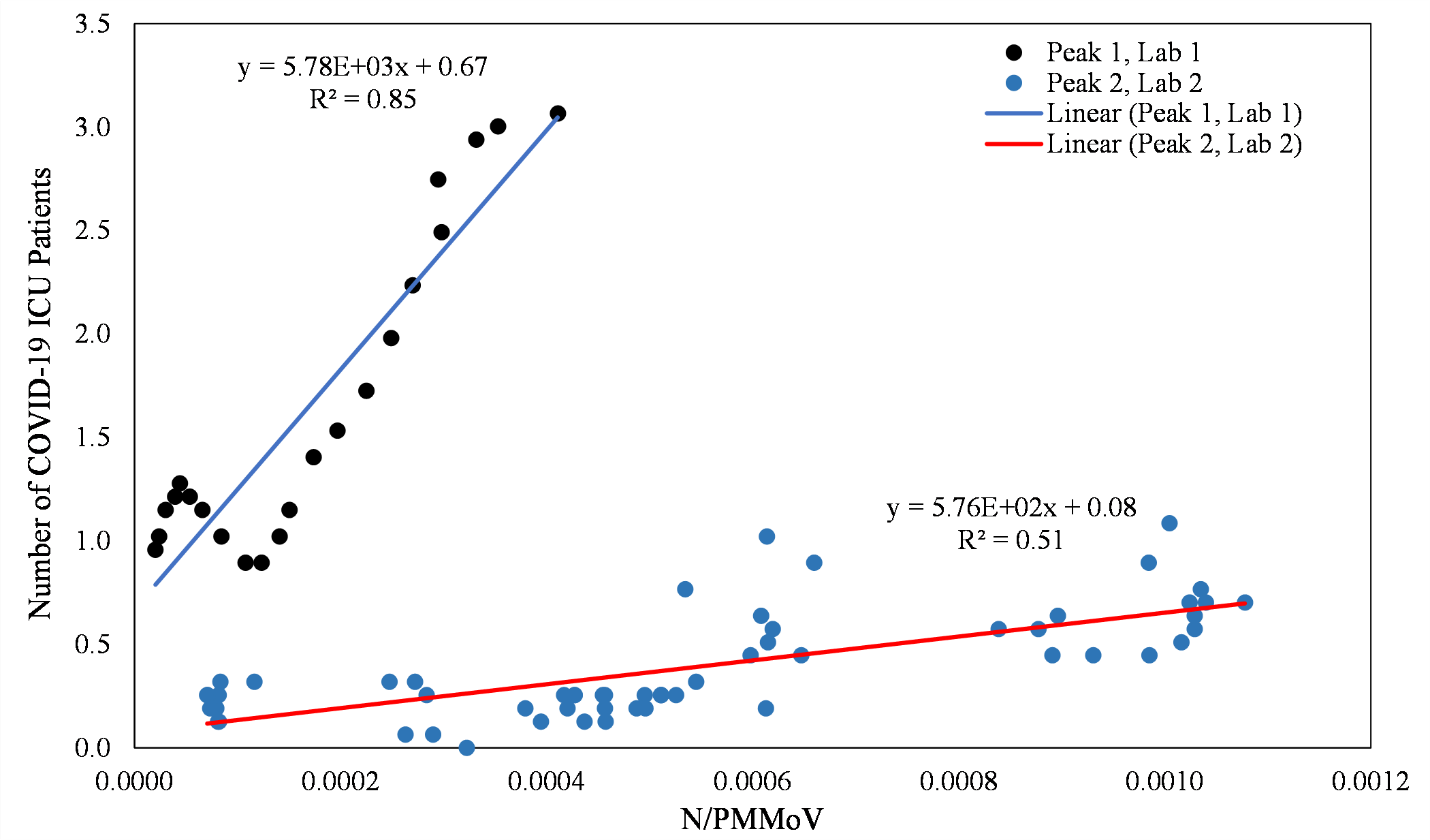


**Figure S18.** Comparison of N/PMMoV for the city of Davis wastewater to Yolo County ICU patients during infection Peak 1 (Lab 1) and Peak 2 (Lab 2).


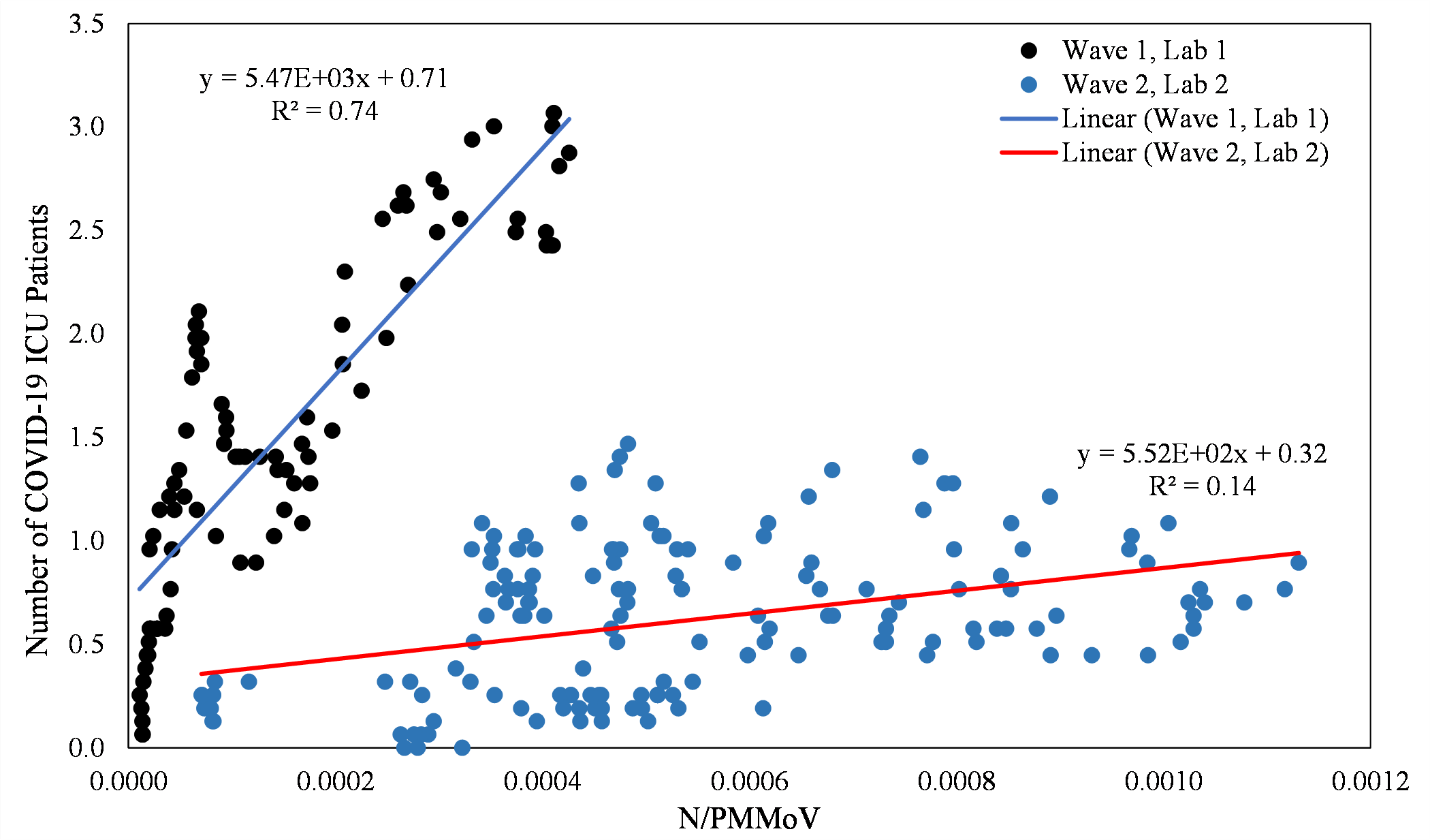


**Figure S19.** Comparison of N/PMMoV for the city of Davis wastewater to Yolo County ICU patients during infection Wave 1 (Lab 1) and Wave 2 (Lab 2).

**Table S6.** Minimum (min.), maximum (max.), average (avg.), and range for hospitalization and ICU admittance per 100k population (pop.) during Wave 1 and Wave 2 for Merced, Stanislaus, and Yolo Counties [23].

| **Time Period** | **County** | **Hospitalization per 100k Pop.** | | | | **ICU per 100k Pop.** | | | |
| --- | --- | --- | --- | --- | --- | --- | --- | --- | --- |
|  |  | **Min** | **Max** | **Avg** | **Range** | **Min** | **Max** | **Avg** | **Range** |
| Wave 1 | Merced | 6.06 | 23.1 | 14.5 | 17.1 | 1.09 | 4.47 | 3.05 | 3.38 |
|  | Stanislaus | 20.2 | 58.1 | 41.1 | 37.9 | 2.39 | 8.84 | 5.98 | 6.45 |
|  | Yolo | 0.96 | 10.9 | 5.20 | 9.90 | 0.06 | 3.07 | 1.51 | 3.00 |
| Wave 2 | Merced | 1.29 | 8.40 | 4.5 | 7.11 | 0.00 | 1.84 | 0.48 | 1.84 |
|  | Stanislaus | 7.27 | 22.2 | 15.1 | 14.9 | 0.25 | 2.77 | 1.46 | 2.52 |
|  | Yolo | 0.51 | 3.58 | 2.24 | 3.07 | 0.00 | 1.47 | 0.62 | 1.47 |

**Table S7.** Percent of the 65+ population fully-vaccinated across all three counties at the start and end of Waves 1 and 2 with the percent increase in vaccination after each wave, and percent of total county population that is 65+ [1, 23].

| **Description** | **Merced** | **Stanislaus** | **Yolo** |
| --- | --- | --- | --- |
| Start Wave 1 (11/30/2021) | 78% | 83% | 93% |
| End Wave 1 (03/01/2022) | 80% | 84% | 95% |
| Percent Change during Wave 1 | 2.7% | 2.4% | 1.4% |
| Start Wave 2 (04/05/2022) | 80% | 85% | 95% |
| End Wave 2 (09/27/2022) | 82% | 87% | 96% |
| Percent Change during Wave 2 | 2.2% | 2.2% | 1.2% |
| Percent of Population that is 65+ | 12% | 14% | 14% |
